# Effects of calcium-containing solutions on fluorescence-detectable plaque area and enamel mineral volume: an in vitro and exploratory clinical study

**DOI:** 10.1186/s12903-026-09789-z

**Published:** 2026-09-15

**Authors:** Zhi-Yun Lin, Earl Fu, Chung-Hsing Li, Ting-Han Chang, Da-Yo Yuh

**Affiliations:** 1 Graduate Institute of Dental Science, College of Oral Medicine, National Defense Medical University, Taipei, Taiwan (R.O.C.); 2 School of Dentistry, College of Oral Medicine, National Defense Medical University, Taipei, Taiwan (R.O.C.); 3 Department of Dentistry, Taipei Tzu Chi Hospital, Buddhist Tzu Chi Medical Foundation, Xindian, New Taipei City, Taiwan (R.O.C.); 4 Division of Periodontology, Tri-Service General Hospital, Taipei, Taiwan; 5 Division of Orthodontic & Pediatric Dentistry, Tri-Service General Hospital, Taipei, Taiwan (R.O.C.); 6 Institute of Oral Biology, College of Dentistry, National Yang Ming Chiao Tung University, Taipei, Taiwan (R.O.C.); 7 Department of Dentistry, College of Dentistry, National Yang Ming Chiao Tung University, Taipei, Taiwan (R.O.C.); 8 Division of Family Dentistry and Oral Diagnosis, College of Oral Medicine, Tri-Service General Hospital and National Defense Medical University, Taipei, Taiwan (R.O.C.); 9 Division of Periodontology, College of Oral Medicine, Tri-Service General Hospital and National Defense Medical University, No.161, Sec. 6, Minquan E. Rd., Neihu Dist., Taipei, 11490 Taiwan (R.O.C.)

**Keywords:** Dental Caries, Thresholded mineralized volume, Calcium compounds, Dental plaque, Exploratory randomized controlled trial

## Abstract

**Background:**

Calcium-containing biomimetic materials have been investigated as adjunctive approaches for managing early enamel demineralization; however, clinical evidence regarding calcium-containing oral sprays remains limited. This study evaluated the effects of calcium-based solutions on fluorescence-detectable plaque-associated area on tooth surfaces through an in vitro experiment and an exploratory randomized clinical trial.

**Methods:**

Extracted teeth with visible enamel carious lesions were immersed for 6 h in ultrapure water, artificial saliva, calcium solution, or artificial saliva plus calcium solution. Threshold-segmented enamel mineralized volume and fluorescence-detectable plaque-associated area were assessed using micro-computed tomography and quantitative light-induced fluorescence (QLF). In an exploratory randomized clinical trial, fifteen children were randomly assigned to placebo, a calcium-containing spray (Formula 1; 0.3% calcium lactate, 0.01% dicalcium phosphate, and 0.01% mesoporous bioactive glass), or the same formulation supplemented with 225 ppm fluoride (Formula 2) for two months. Fluorescence-detectable plaque-associated area and salivary calcium concentration were evaluated.

**Results:**

In vitro, threshold-segmented enamel mineralized volume showed numerically higher post-treatment values in the artificial saliva and calcium-containing solution groups, while QLF-detected plaque-associated fluorescent area decreased across groups. Changes in mineralized volume were significantly negatively correlated with changes in fluorescence-detectable plaque-associated area (Spearman’s ρ = −0.771, *P* < 0.0001). *Clinically*, salivary calcium increased in both calcium-containing spray groups, although within-group changes did not reach statistical significance (both *P* = 0.0625). The calcium-plus-fluoride comparator demonstrated a significantly greater increase in Δsalivary calcium than placebo (*P* < 0.05). Median fluorescence-detectable plaque-associated area decreased from 5.47% to 2.33% in Formula 1 and from 8.63% to 3.06% in Formula 2, whereas the placebo group showed minimal change (2.42% to 2.76%). Exploratory ANCOVA adjusted for baseline QLF, age, and sex supported a significant overall treatment effect (*P* = 0.009).

**Conclusions:**

Both calcium-containing oral sprays were associated with greater reductions in the primary clinical outcome, fluorescence-detectable plaque-associated area, compared with placebo. No statistically significant difference in the primary outcome was observed between Formula 1 and Formula 2. Complementary in vitro findings showed numerically higher post-treatment mineralized volume values and a significant inverse association between ΔMV and ΔQLF-detected fluorescent area under controlled conditions. Together, these exploratory findings support further investigation of calcium-containing oral sprays in larger, adequately powered clinical trials.

**Clinical significance:**

Calcium-containing sprays may have potential as an adjunctive home-care approach for reducing fluorescence-detectable plaque-associated signals in children with early enamel demineralization.

**Clinical trial registration:**

NCT07269730, retrospectively registered on November 25, 2025. URL https//clinicaltrials.gov/study/NCT07269730.

**Supplementary Information:**

The online version contains supplementary material available at https://doi.org/10.1186/s12903-026-09789-z.

## Introduction

Dental caries is the most prevalent dental disease worldwide, primarily caused by cariogenic bacteria such as *Streptococcus mutans* [1]. These bacteria metabolize dietary sugars into acids that demineralize tooth enamel. Thus, caries lesions develop as a consequence of an imbalance between demineralization and remineralization processes. [2]. Plaque accumulation initiates enamel demineralization [3], forming porous white spot lesions with an opaque appearance. In contrast, remineralization redeposits minerals into enamel and may reverse early carious lesions [4, 5].

Fluoride remains one of the most established agents for promoting enamel remineralization [6, 7]. Although fluoride remains highly effective, concerns regarding excessive fluoride exposure and its potential adverse effects have encouraged interest in adjunctive remineralization strategies that incorporate calcium- and phosphate-based materials [8]. Calcium and phosphate ions play essential roles in enamel mineral balance and have been incorporated into a variety of preventive and remineralization-oriented products [9–11]. Studies have shown enhanced enamel remineralization with calcium-containing chewing gum [12], casein phosphopeptide-amorphous calcium phosphate paste [13], and hydroxyapatite toothpaste [14]. Moreover, in a randomized clinical trial, combining functionalized tricalcium phosphate with fluoride improved caries arrest in preschool children compared with fluoride varnish alone [15].

Although these calcium-containing products have shown promising results, most currently available formulations are delivered as toothpastes, topical pastes, varnishes, or chewing gums. As maintaining oral hygiene in young children remains challenging, spray-based delivery systems may provide a more convenient approach for oral care and caregiver-assisted administration in pediatric populations [16]. Quantitative light-induced fluorescence (QLF) has recently been demonstrated to provide moderate-to-high diagnostic accuracy for caries detection and represents a reliable non-ionizing adjunct for monitoring changes in fluorescence-associated caries lesions [17]. However, clinical evidence regarding calcium-containing oral sprays remains limited, and their potential effects on plaque-associated fluorescence and enamel mineralization-related outcomes remain unclear.

Therefore, the primary objective of this exploratory clinical study was to evaluate whether calcium-containing oral sprays influence fluorescence-detectable plaque-associated area measured by QLF. Secondary objectives included assessment of salivary calcium concentration and exploratory evaluation of plaque microbiota. In addition, an in vitro experiment was conducted to investigate changes in threshold-segmented enamel mineralized volume and fluorescence-detectable plaque-associated area under controlled conditions. We hypothesized that calcium-containing solutions would be associated with measurable changes in threshold-segmented enamel mineralized volume and reduced QLF-detected fluorescent area under controlled in vitro conditions, and that calcium-containing oral sprays would reduce fluorescence-detectable plaque-associated area and increase salivary calcium concentration compared with placebo in the exploratory clinical trial.

## Materials and methods

### In vitro experiment

#### Sample processing and grouping

Twenty extracted human third molars exhibiting visible occlusal enamel carious lesions were obtained from de-identified specimens collected under approval from the Tri-Service General Hospital Institutional Review Board (TSGHIRB No. A202105108). Donor age information was not available for the de-identified extracted teeth. After cleaning with deionized water and drying, the teeth were randomly divided into four groups (*n* = 5/group): ultrapure water (control), artificial saliva (AS), calcium solution, and AS plus calcium solution (1 :1 ratio). The calcium solution used in the in vitro experiment was the commercially available Kids Oral Spray (Toothfilm Inc., Taipei, Taiwan; Lot No. JG04), containing 0.3% calcium lactate, 0.01% dicalcium phosphate, and 0.01% mesoporous bioactive glass. After initial examinations, each tooth was soaked in the designed solution in a 50mL centrifuge tube for 6 h and then dried for post-treatment examinations. Artificial saliva was prepared following a previous study [18], consisting of 0.1029 g CaCl₂·2 H₂O, 0.04066 g MgCl₂, 0.544 g KH₂PO₄, 4.766 g HEPES buffer acid form, and 2.2365 g KCl in 1000 mL distilled water (pH 7).

### In vitro experimental procedures

Caries lesions on the tooth surfaces were identified using a scanning electron microscope (SEM) and then examined three-dimensionally *via* micro-computed tomography (µ-CT) in sagittal, coronal, and horizontal views. Additionally, residual plaque-associated deposits on the extracted tooth surfaces were visualized using quantitative light-induced fluorescence (QLF). These analyses allowed for the quantification of threshold-segmented enamel mineralized volume and plaque-associated fluorescence-detectable areas before and after treatment with the four solutions. All teeth were examined with µ-CT and QLF before and after treatments.

### Quantitative light-induced fluorescence (QLF)

Residual plaque-associated deposits on extracted tooth surfaces were visualized using QLF (Metal Industries Research & Development Centre, Kaohsiung, Taiwan). Three types of images were captured: (1) an original photograph (2), a binary plaque image, and (3) a fluorescence image of plaque-associated red fluorescence. We defined the same region of interest on the before and after QLF fluorescence image using ImageJ. Through the images, the relative fluorescence-detectable plaque-associated area was quantified as the percentage of QLF-observed plaque-associated red fluorescence within the total region of interest (Fig. 2C), while the difference in the areas before and after treatment was recorded as ΔQLF-area [19].

### Scanning electron microscopy and micro-computed tomography

Caries lesions were examined using scanning electron microscopy (SEM; Apreo 2 S, Thermo Fisher Scientific, Waltham, MA, USA) at 130× magnification and 5.0 kV. Micro-computed tomography (micro-CT) images were acquired using a Quantum GX2 micro-CT system (PerkinElmer, Waltham, MA, USA) at 90 kV and 160 µA, with a 4.5-min scan time. The reconstructed DICOM image stacks had a matrix size of 512 × 512 pixels, with a pixel spacing of 0.05 × 0.05 mm and a slice thickness of 0.05 mm. Therefore, the reconstructed voxel size was 50 μm isotropic, as determined from the DICOM metadata.

The reconstructed DICOM image stacks were imported into Materialise Mimics software for segmentation and volumetric analysis. For each tooth, the whole enamel region was selected as the region of interest, rather than a lesion-specific region. A gray-value threshold of approximately 4000, with the upper threshold set to the maximum gray value, was used to segment the mineralized enamel region. When minor threshold adjustment was required among different teeth, the same threshold was maintained for the paired pre- and post-treatment scans of the same tooth to ensure within-tooth longitudinal consistency. The thresholded mask was converted into a three-dimensional object, and the threshold-segmented enamel mineralized volume was calculated in mm³. After alignment of the paired pre- and post-treatment datasets based on tooth morphology, Boolean operations were applied to the paired 3D objects to estimate the volume difference before and after treatment. This value was recorded as the change in threshold-segmented enamel mineralized volume (ΔMV) [20].

### Exploratory randomized clinical trial

#### Experimental design and patient selection

**Fig. 1:**
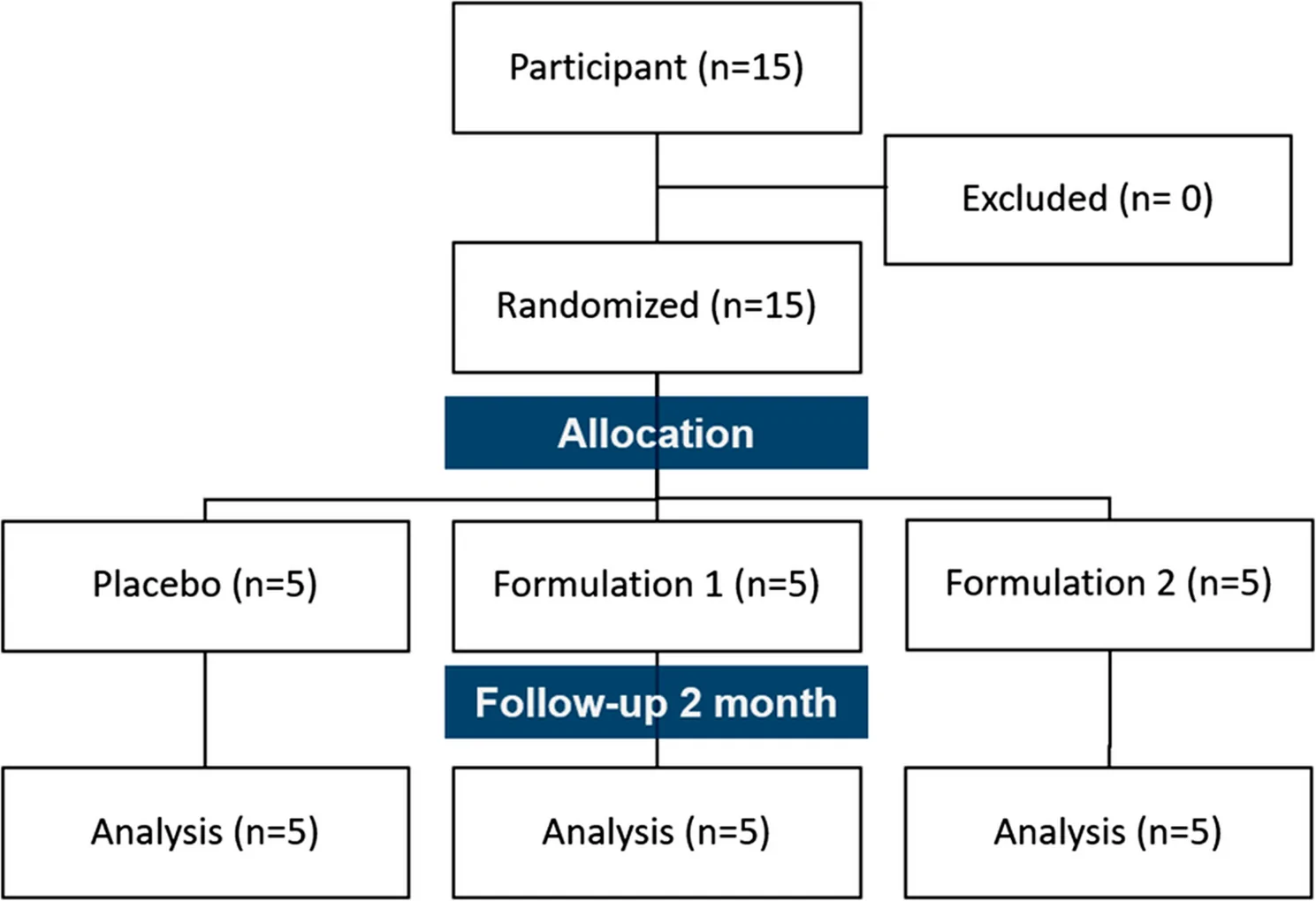
CONSORT flow diagram showing enrolment, allocation, follow-up, and analysis of participants (*n* = 15). Participants were randomized to placebo (*n* = 5), Formulation 1 (*n* = 5), or Formulation 2 (*n* = 5), with no losses or exclusions

The exploratory randomized clinical trial (RCT) was approved by the Tri-Service General Hospital Institutional Review Board (TSGHIRB No. A202305084). A double-blind RCT was conducted with 15 children presenting early carious lesions (Fig. 1). Recruitment began after IRB approval on June 11, 2023 and was completed on July 31, 2024. Inclusion criteria required participants to have an ICDAS code ≥ 1 and ≤ 3 as determined by a single experienced examiner (Dr. CHL). The mean age of the participants was 6.26 ± 3.0 years. The random allocation sequence was generated by an independent researcher using a computer-generated random number table. Participants were randomly assigned to one of three groups: placebo (baseline control), calcium-containing spray (Formulation 1), and calcium plus fluoride (Formulation 2, calcium-plus-fluoride comparator). Eligibility was determined by the enrolling clinician, whereas group assignment was conducted by an independent researcher using sequentially numbered, opaque, sealed envelopes to ensure allocation concealment. Participants, caregivers, and outcome assessors were blinded to group allocation. All sprays were packaged in identical bottles with standardized appearance and labeling, and were coded by an independent researcher (Ms. ZYL) to maintain blinding until data analysis was completed.

Formulation 1 consisted of the commercially available calcium-containing spray described above (0.3% calcium lactate, 0.01% dicalcium phosphate, and 0.01% mesoporous bioactive glass), whereas Formulation 2 consisted of the same formulation supplemented with 225 ppm fluoride (0.05% NaF). The pH values of the experimental solutions and spray formulations were measured using a pH meter before use and ranged from 5.5 to 6.5. Differences before and after treatment were analyzed. Plaque and saliva samples were collected from all participants. Unstimulated whole saliva (approximately 1 mL) was collected by direct expectoration into a 15-mL centrifuge tube without the use of any specified salivary stimulation procedure and was temporarily stored on ice at approximately 4 °C before processing. All procedures were conducted following ethical guidelines and regulations, with informed consent obtained from all participants (TSGHIRB No: A202305084 and NCT07269730). All participants were provided with the same commercially available fluoride-free toothpaste (Toothfilm Inc., Taipei, Taiwan) and were instructed to brush twice daily under caregiver supervision throughout the study period. According to the manufacturer, the toothpaste was formulated with glycerin, water, silica, xylitol, calcium carbonate, dicalcium phosphate, and calcium lactate and did not contain fluoride. Standardized toothbrushing instructions were given to all participants, including using an age-appropriate amount of toothpaste, positioning the toothbrush at approximately 45° to the gingival margin, gently brushing all tooth surfaces, and brushing for approximately two minutes each time. Participants were asked to maintain this standardized oral hygiene routine during the two-month intervention. No restrictions regarding eating or drinking after spray application were imposed.

### Experimental procedures

At the initial clinic visit, plaque and saliva samples were collected. Participants were instructed on product use, and follow-up visits ensured compliance. Participants used the assigned spray twice daily (morning and evening) after brushing teeth under caregiver supervision. The spray was pressed 2–3 times (0.4mL-0.6mL) each time. Adherence was verbally confirmed at every two-week follow-up visit, with no deviations from the intended intervention reported. Harms, defined as any unexpected oral symptoms such as tooth sensitivity, gingival irritation, or mucosal discomfort, were systematically assessed at each visit through clinician questioning and oral examination. After two months, dental conditions were assessed using QLF, and additional plaque and saliva samples were collected.

In the clinical trial, QLF imaging produced three outputs: (1) an original photograph (3), a binary image highlighting carious lesions, and (3) a fluorescent image. The QLF-area (%) and its change (ΔQLF-area) were measured and compared.

### 16 S rRNA gene sequence analyses

To examine bacterial changes in plaque before and after calcium spray use, three out of 15 participants were selected for bacterial sampling (pre- and post-treatment). Plaque samples were collected using a periodontal probe and stored in 1.5 mL tubes containing 500 µL of preservation solution at -80 °C.

Bacterial DNA was extracted following the manufacturer’s instructions using the EasyPure Bacteria Genomic DNA Kit (TransGen Biotech, Cat No. EE161). DNA purity and concentration were verified using a Nanodrop spectrophotometer. The V3 and V4 regions of the 16 S rRNA gene were amplified with primers (CCTACGGRRBGCASCAGKVRVGAAT and GGACTACNVGGGTWTCTAATCC) and the MetaVX Library Preparation Kit. PCR products (~ 600 bp) were confirmed via 1% agarose gel electrophoresis. Sequencing reads were processed using QIIME2, with quality filtering, denoising, and operational taxonomic unit (OTU) clustering at a 97% similarity threshold. Taxonomic classification was performed using the Silva 138 reference database and the Ribosomal Database Project (RDP).

### Salivary calcium content analyses

Saliva samples collected during the clinical trial were centrifuged at 3000 rpm for 10 min to obtain the clarified supernatant, which was then diluted tenfold for calcium measurement. Calcium concentration was determined using an arsenazo III colorimetric assay and measured spectrophotometrically at 652 nm with a microplate reader [21].

### Statistical analysis

Data aggregation and preliminary calculations were performed using Excel 2013. Statistical analyses were conducted using GraphPad Prism 9.5 and SPSS version 20. Given the small sample size, non-parametric methods were used for the primary analyses. Within-group before–after comparisons were assessed using the Wilcoxon matched-pairs signed-rank test. Between-group differences in change scores, calculated as post-treatment minus baseline values, were assessed using the Kruskal-Wallis test followed by Dunn’s multiple comparisons test. Associations between changes in mineral volume (MV) and QLF-area were evaluated using Spearman’s rank correlation; fitted lines were shown only to visualize the direction of the associations and were not used for statistical inference.

ANCOVA was performed as an exploratory sensitivity analysis for post-treatment QLF outcomes, with treatment group as the fixed factor and baseline QLF, age, and sex as covariates. All randomized participants were included in the final analysis, and no imputation was required because there was no missing data. A two-sided p value < 0.05 was considered statistically significant. No formal a priori power-based sample-size calculation was performed because the clinical component was designed as an exploratory pilot study. Exploratory post-hoc power analyses based on change scores were performed using G*Power 3.1.9.7 and are reported only as supplementary information for planning future confirmatory studies.

## Results

### In vitro experiment

**Fig. 2:**
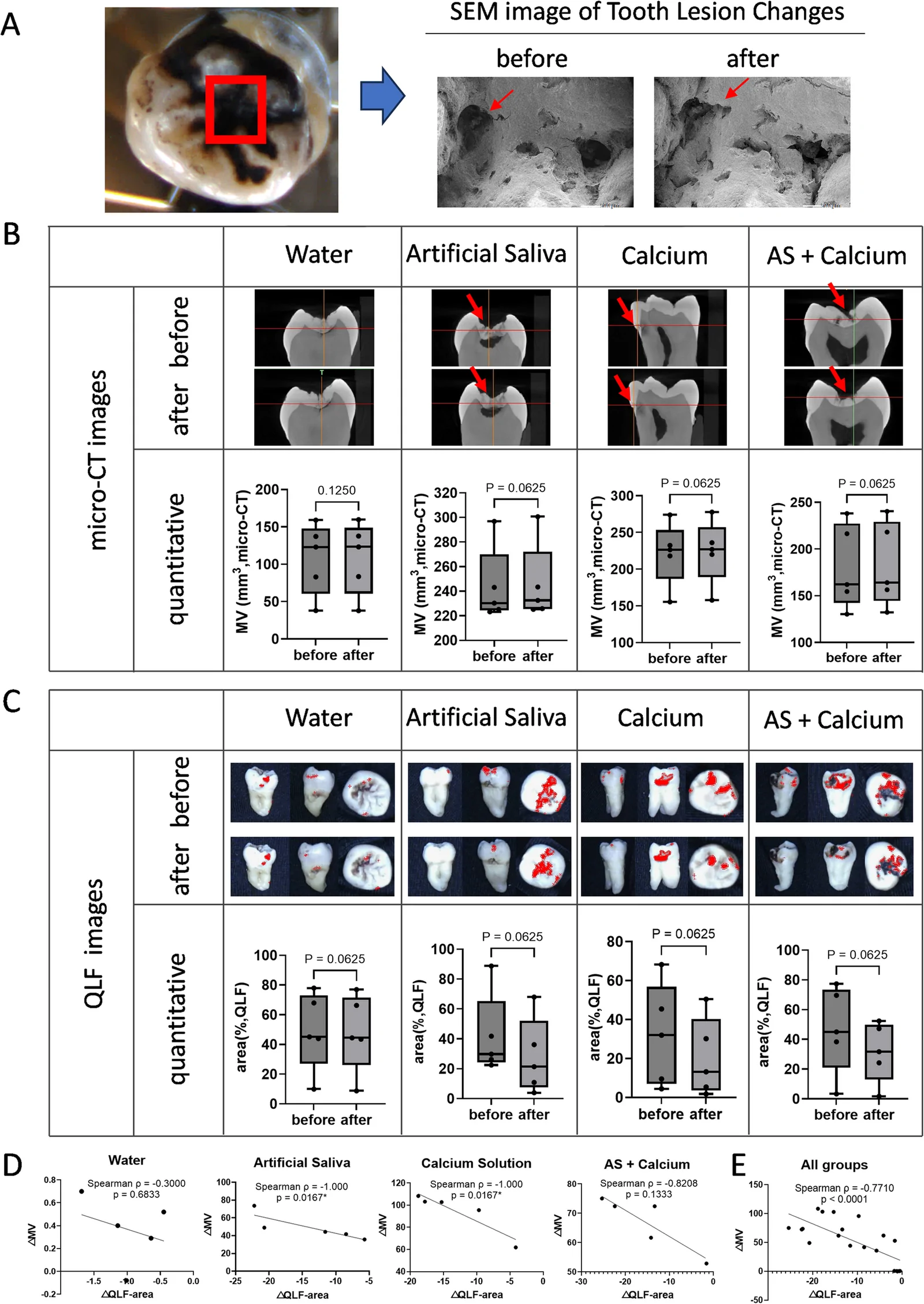
In vitro effects of calcium solutions on QLF-area and MV changes. **A** SEM images of tooth occlusal lesions before and after calcium solution treatment (Red box and blue arrow indicate the area where the 130× magnification pictures are shown; Red arrows indicate the lesion with the demineralization). **B** Micro-CT images of teeth in four solution groups, while the threshold-segmented enamel mineralized volumes (MVs) were analyzed and compared quantitatively before and after rinsing (Red arrows indicate the demineralization lesion; AS + Calcium: the group of combination of artificial saliva and calcium solution). **C** QLF images of teeth in four groups, with quantitative analysis of QLF-areas (Red fluorescence indicates plaque-associated fluorescence detected by QLF). (Box plots indicate the median, interquartile range, whiskers (minimum to maximum), and individual data points; *n* = 5 per group; Wilcoxon matched-pairs signed-rank test in **B** and **C**). **D** Correlation between ΔQLF-areas and ΔMVs across groups (Spearman’s rank correlation. *n* = 5 per group; AS + Calcium: the group of combination of artificial saliva and calcium solution). **E** Correlation between ΔQLF-area and ΔMV across all in vitro treatment groups. The association was assessed using Spearman’s rank correlation. Individual data points are shown; *n* = 20 total. Spearman’s ρ = −0.7710, *p* < 0.0001. The fitted lines in **D** and **E** are shown only to visualize the direction of the association

Tooth surfaces were examined using SEM, and the changes before and after treatment were clearly observed (Fig. 2A). The µ-CT was used for quantitative measurement of threshold-segmented enamel mineralized volume. Threshold-segmented enamel mineralized volume showed little apparent change in the water group and numerically higher post-treatment values in the artificial saliva, calcium solution, and artificial saliva plus calcium solution groups; however, the within-group changes did not reach statistical significance (Fig. 2B). QLF analysis showed lower post-treatment QLF-detected fluorescent areas across all groups, including the water group (Fig. 2C). Across all in vitro specimens, ΔMV was significantly negatively correlated with ΔQLF-detected fluorescent area (Spearman’s ρ = −0.771, *P* < 0.0001; Fig. 2E). Group-specific analyses showed negative associations in the artificial saliva and calcium-containing groups but not in the water group (Fig. 2D).

### Exploratory randomized clinical trial

#### Changes in dental plaque composition after calcium-spray use

**Fig. 3:**
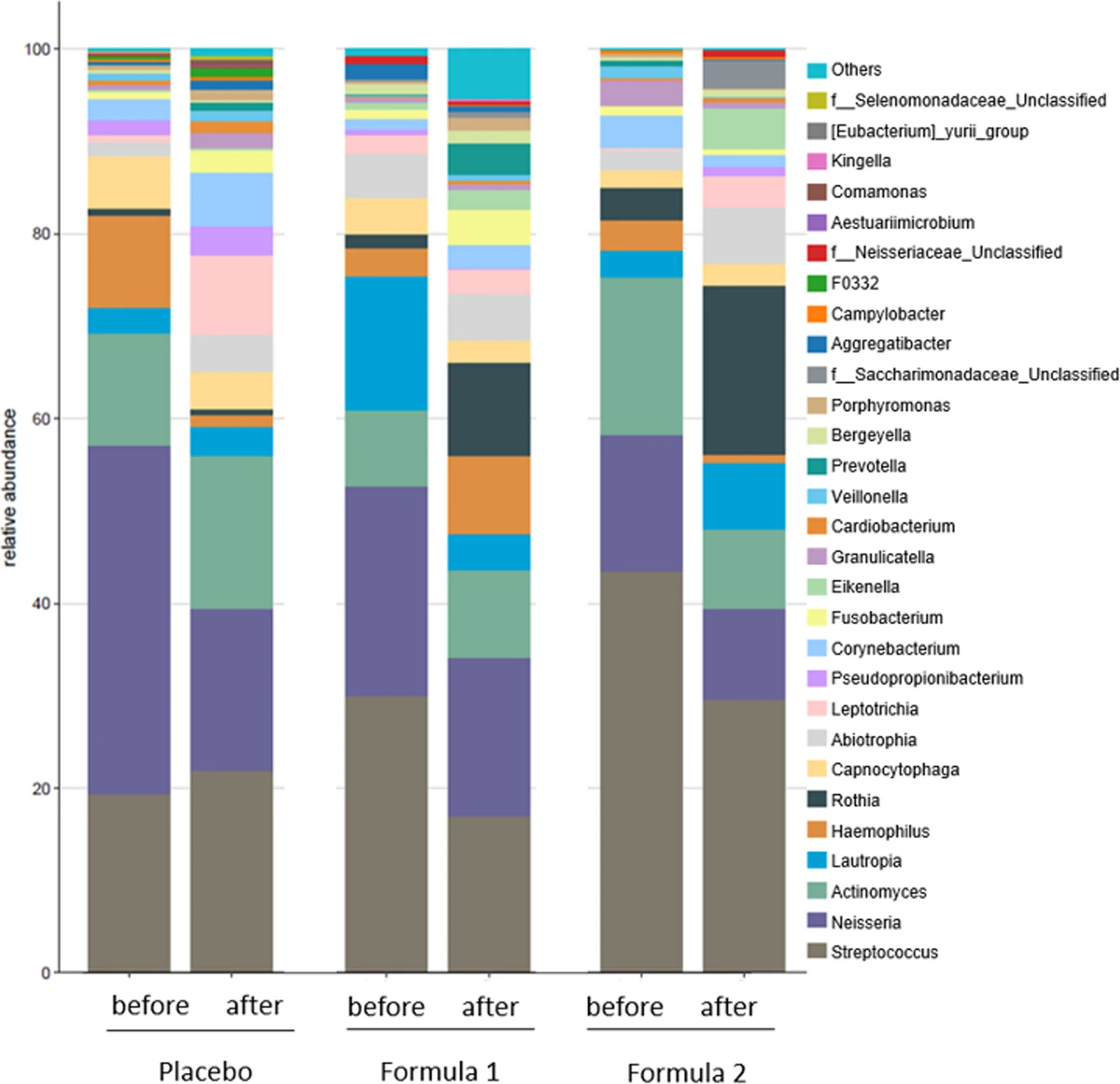
Effects of calcium sprays on *Streptococcus* abundance in dental plaque microbiota. The 16 S rRNA sequencing of dental plaque samples before and after 2-month treatments of three oral spray formulations (6 samples, paired before/after, red arrowhead indicates *streptococcus*)

16 S rRNA sequencing identified 30 bacterial genera in plaque samples (Fig. 3). Descriptive differences in bacterial composition were observed between baseline and two months across the three groups. Streptococcus proportions remained stable in the placebo group (19.41% before vs. 21.91% after) but decreased in the calcium spray groups—Formulation 1 (29.99 vs. 17.03%) and Formulation 2 (43.52 vs. 29.55%). Meanwhile, the relative abundance of Actinomyces slightly increased in the placebo group (12.06% vs. 16.47% after), remained stable in Formulation 1 (8.21 vs. 9.42%) and decreased in Formulation 2 (17.04% vs. 8.62%). Descriptively, the combined relative abundance of Streptococcus and Actinomyces decreased from 38.2% to 26.45% in Formula 1 and from 60.56% to 38.17% in Formula 2. Because of the very limited sample size, these observations are descriptive only.

### Changes in saliva calcium levels after calcium-spray use

**Fig. 4:**
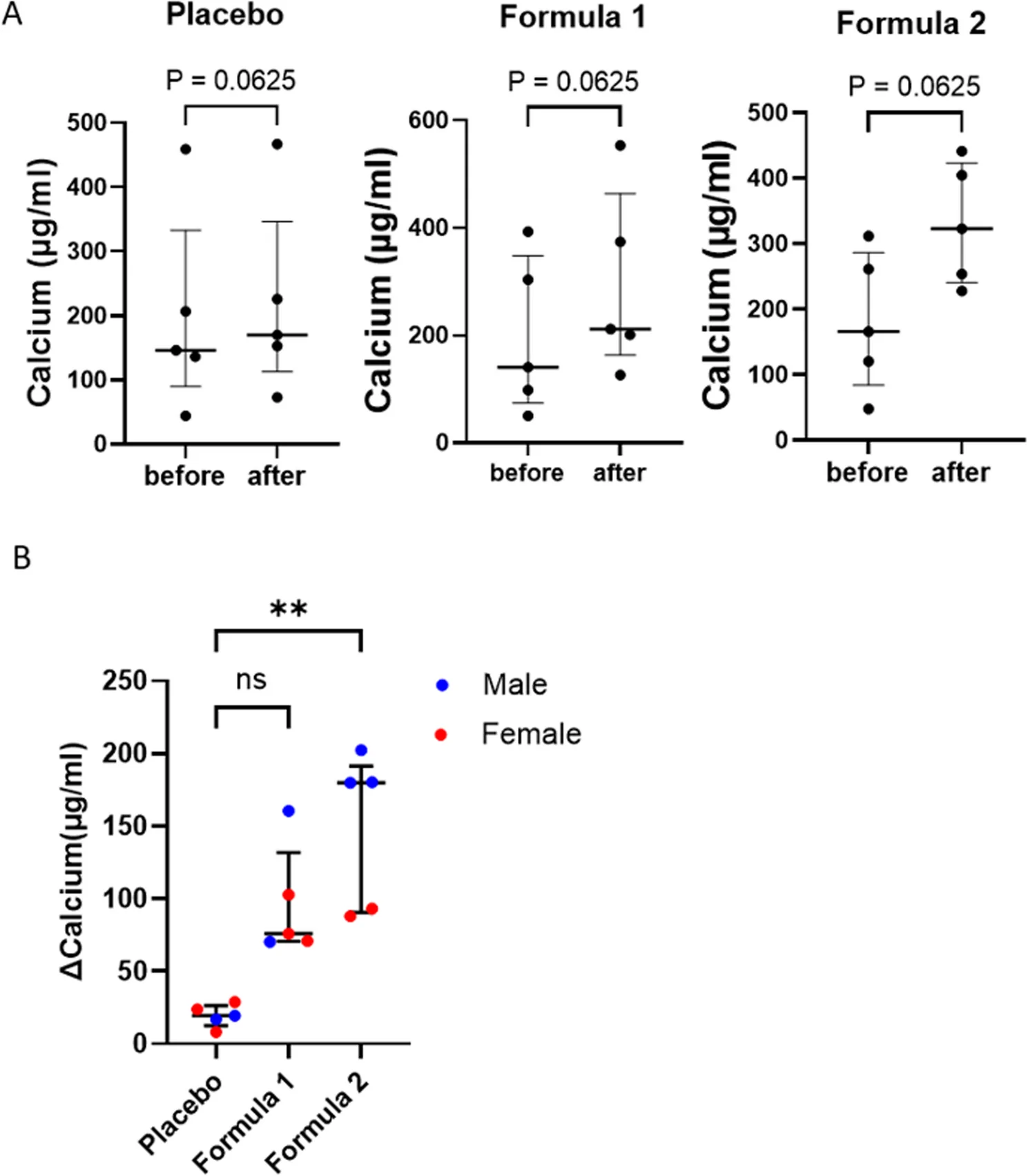
Effects of calcium sprays on salivary calcium levels in the pilot clinical trials. **A** Salivary calcium levels before and after using the placebo, calcium-containing formulation (Formula 1), and calcium-plus-fluoride formulation (Formula 2) (Box plots indicate the interquartile range, mean, and median, with individual data points shown as dots; *n* = 5 per group, within-group comparisons were performed using the Wilcoxon matched-pairs signed-rank test.). **B** Changes in the salivary calcium levels before and after using sprays among the patients with placebo and two calcium groups (Formula 1 and Formula 2) for 2 months. Data are shown as median with interquartile range, with individual data points shown as dots; *n* = 5 per group. Between-group comparisons were performed using the Kruskal-Wallis test followed by Dunn’s multiple comparisons test comparing each formulation group with the placebo group. ns : not significant, **p < 0.01

Salivary calcium levels showed non-significant within-group increases after treatment in all groups (Fig. 4A). Between-group comparison of Δsalivary calcium showed that the increase was significantly greater in the calcium-plus-fluoride comparator group than in the placebo group, whereas the calcium-containing formulation group did not differ significantly from placebo (Fig. 4B).

### Reduction in fluorescence-detectable plaque-associated area after calcium spray

**Fig. 5:**
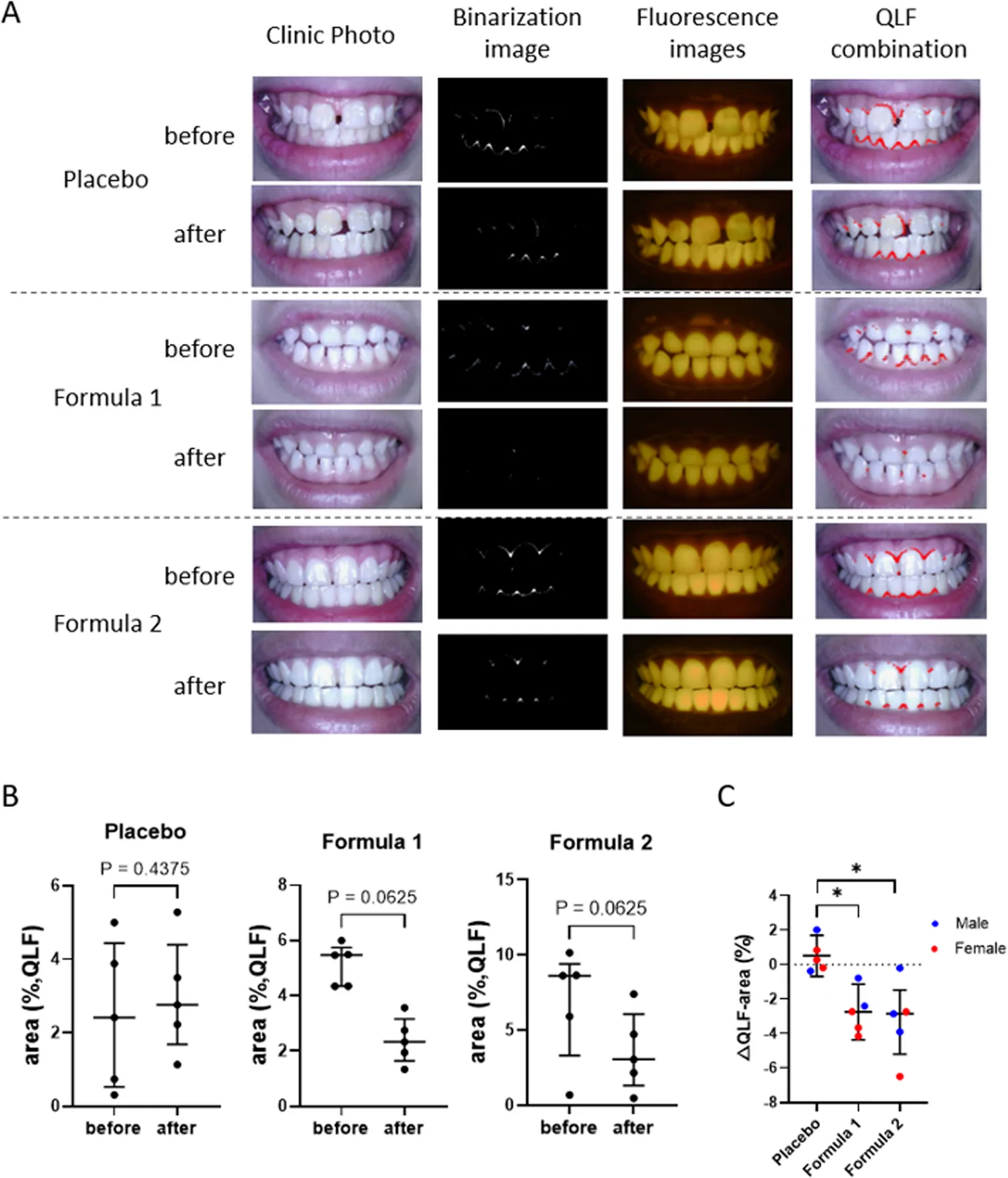
Effects of calcium-sprays on QLF-detected plaque-associated fluorescence in the clinical trial. **A** Representative clinical and QLF images before and after the 2-month intervention. Images were processed using a combination of clinical photography (pictures in the left column) and binarized QLF images, where the red fluorescence indicated plaque-associated fluorescence detected by QLF (pictures in the right column). Formulations included the placebo, calcium spray (Formula 1), and calcium spray with fluoride (Formula 2). The representative cases were selected from participants aged 12 years (Placebo), 6 years (Formula 1), and 13 years (Formula 2). **B** Comparison of QLF-detected plaque areas before and after using the sprays within each of the test groups. Data are shown as median with interquartile range, with individual data points shown as dots; *n* = 5 per group, within-group comparisons were performed using the Wilcoxon matched-pairs signed-rank test. **C** Quantitative changes in ΔQLF-area across groups. Data are shown as median with interquartile range, with individual data points shown as dots; *n* = 5 per group; *: significant difference at *p* < 0.05 by Kruskal-Wallis test followed by Dunn’s multiple comparisons test

The clinical photographs in Fig. 5 show QLF results, where red fluorescence on the tooth surfaces in the left column of QLF combinations indicates fluorescence-detectable plaque-associated areas (Table 1 and Supplementary Figure). After two months of intervention, fluorescence-detectable plaque-associated areas were reduced in both calcium-containing spray groups. Results are presented as median (interquartile range, IQR). The calcium-containing group (Formulation 1) showed a decrease in QLF-area from a median of 5.47% (IQR, 4.35 to 5.75) before treatment to 2.33% (IQR, 1.64 to 3.16) after treatment; calcium-plus-fluoride comparator group (Formulation 2) showed a decrease in QLF-area from a median of 8.63% (IQR, 3.31 to 9.41) before treatment to 3.06% (IQR, 1.33 to 6.07) after treatment. In contrast, the placebo group showed only minimal change in QLF-area, from a median of 2.42% (IQR, 0.53 to 4.44) before treatment to 2.76% (IQR, 1.68 to 4.39) after treatment, with a median change of 0.28% (IQR, − 0.28 to 1.43) (Fig. 5A–B). The ΔQLF-area differed among groups, with median changes of 0.28% (IQR, − 0.28 to 1.43) in the placebo group, − 2.72% (IQR, − 3.91 to − 1.60) in the calcium-containing group (Formulation 1), and − 2.86% (IQR, − 5.20 to − 1.48) in the calcium-plus-fluoride comparator group (Formulation 2) (Fig. 5C). The ΔQLF-areas in both calcium-containing formulations were significantly lower than those in the placebo group. No adverse events were observed in any group throughout the study.

**Table 1:** Baseline characteristics and QLF/ICDAS outcomes of clinical participants

Case	Sex	Age	Group	QLF-area, %		ICDAS code	
				Before	After	Before	After
1	Female	6	Placebo	0.31	1.14	1	1
2	Male	4	Placebo	0.74	2.76	1	1
3	Female	4	Placebo	5	5.28	1	1
4	Male	12	Placebo	3.88	3.5	1	1
5	Female	8	Placebo	2.416	2.227	1	1
6	Female	6	Formulation 1	5.49	1.338	2	1
7	Female	6	Formulation 1	5.47	2.75	2	1
8	Female	6	Formulation 1	6	2.33	1	1
9	Male	8	Formulation 1	4.35	3.56	1	1
10	Male	8	Formulation 1	4.344	1.94	1	1
11	Male	4	Formulation 2	0.69	0.48	1	1
12	Male	13	Formulation 2	5.92	3.06	1	1
13	Female	4	Formulation 2	8.66	2.17	1	1
14	Female	3	Formulation 2	10.15	7.41	1	1
15	Male	8	Formulation 2	8.63	4.73	1	0

As an exploratory sensitivity analysis, ANCOVA was performed for post-treatment QLF outcomes after adjustment for baseline QLF, age, and sex. Treatment group remained associated with post-treatment QLF after adjustment (F = 8.203, *p* = 0.009; Supplementary Table 1). Adjusted estimated marginal means were lower in Formula 1 and Formula 2 than in placebo, with adjusted mean ± SE values of 2.46 ± 0.46 and 1.35 ± 0.58, respectively, compared with 4.99 ± 0.57 in the placebo group (both *p* < 0.01; Supplementary Table 2).

Exploratory post-hoc power analysis based on change scores showed large observed effect sizes for Formula 1 versus placebo and Formula 2 versus placebo, with Cohen’s d values of 2.846 and 2.153 and observed powers of 0.915 and 0.845, respectively. The overall one-way ANOVA showed Cohen’s f = 1.035 and an observed power of 0.892 (Supplementary Table 3). These estimates were used only to inform sample size planning for future confirmatory studies.

## Discussion

This study assessed the effects of calcium-containing oral sprays on thresholded enamel mineral volume in vitro and fluorescence-detectable plaque area in an exploratory clinical trial. Early caries detection is essential for prevention. Although radiography remains the diagnostic standard [22], it is less suitable for children [23]. Noninvasive tools such as QLF permit early detection by visualizing red fluorescence from bacterial porphyrins, which reflects biofilm acidogenicity [24–26]. µ-CT provides quantitative mineralization data but is limited to in vitro use [27]. The in vitro and clinical components of this study were designed to address different but complementary questions. Because repeated micro-CT assessment of enamel mineral changes is not feasible in children due to radiation exposure, the in vitro experiment was conducted under controlled conditions to examine whether changes in fluorescence-detectable plaque-associated area occurred concurrently with threshold-segmented enamel mineralized volume changes. The clinical trial then employed QLF as a non-invasive imaging modality to longitudinally monitor plaque-associated fluorescence following calcium spray application. Accordingly, the in vitro findings should not be interpreted as direct validation of the clinical outcomes but rather as complementary observations obtained under controlled experimental conditions.

Using µ-CT and QLF, our in vitro results showed a trend toward higher threshold-segmented enamel mineralized volume following 6-hour immersion in artificial saliva or calcium-containing solutions and a trend toward lower fluorescence-detectable plaque-associated deposits under controlled conditions (Fig. 2). A significant negative correlation was observed between ΔMV and ΔQLF-detected fluorescent area across all in vitro specimens, indicating that the two measured parameters changed in opposite directions under the experimental conditions (Fig. 2E). This association should not be interpreted as evidence of causality, absolute mineral gain, or enamel remineralization. By contrast, water immersion also reduced the QLF-detected fluorescent area without an apparent corresponding change in threshold-segmented mineralized volume, suggesting that part of the fluorescence reduction may reflect removal of loosely adherent surface deposits during immersion rather than a treatment-specific chemical effect. One limitation of the micro-CT analysis is that hydroxyapatite phantom calibration and study-specific beam-hardening correction were not performed. Therefore, the gray values were not converted into absolute mineral density values. The micro-CT findings should accordingly be interpreted as threshold-segmented volumetric changes within the whole enamel region, rather than as absolute mineral density, mineral content, lesion reversal, or direct evidence of remineralization.

In the present pilot clinical trial, descriptive reductions in the relative abundance of Streptococcus were observed on both the calcium-containing spray and calcium-plus-fluoride comparator spray (Formulations 1 and 2), whereas minimal changes were observed in the placebo group (Fig. 3). The 16 S rRNA microbiome analysis was based on a very limited number of participants and should therefore be considered descriptive and hypothesis-generating only. These data are insufficient to support definitive conclusions regarding changes in microbial composition or cariogenic dysbiosis. Nevertheless, this pilot finding is consistent with studies linking caries progression to oral microbial dysbiosis [28, 29]. During caries development, frequent sugar exposure increases acid production, favoring acidogenic and aciduric bacteria such as *Streptococcus mutans* while suppressing acid-sensitive species [30]. Although this study did not investigate the mechanisms by which calcium influences plaque composition, prior studies suggest that calcium may contribute to maintaining microbial balance in the oral environment [31]. In the present trial, salivary calcium levels showed non-significant within-group increases after spray use, while between-group analysis suggested a greater Δsalivary calcium in the calcium-plus-fluoride comparator group than in the placebo group (Fig. 4). The elevated salivary calcium observed here may therefore support conditions favorable for potential enamel remineralization [32, 33]. Previous studies have reported potential benefits of combining calcium- and phosphate-containing systems with fluoride for enamel mineralization [34, 35]. However, in the present exploratory trial, no statistically significant difference in the primary clinical outcome was observed between the calcium-containing formulation and calcium-plus-fluoride formulation.

Previous work suggests that calcium may affect cariogenic biofilms not only through remineralization, but also through modulation of acidogenicity. Calcium glycerophosphate has long been discussed as an anticaries agent with possible plaque pH-buffering effects, and more recent biofilm studies have shown that calcium-containing formulations can increase biofilm pH and calcium availability after sucrose challenge [36]. Consistent with this concept, recent reviews have proposed that calcium-based materials may help neutralize bacterial acids and create a less demineralizing plaque environment [11]. However, because plaque pH and acid production were not directly measured in our study, this potential mechanism should be interpreted cautiously [37].

Recent evidence has demonstrated that QLF provides clinically acceptable diagnostic accuracy for detecting carious lesions and can serve as a non-ionizing adjunctive imaging modality. Therefore, QLF was selected in the present study as a quantitative tool to longitudinally monitor changes in fluorescence-detectable plaque-associated areas rather than as a definitive diagnostic method for enamel remineralization [11]. Because the participants were young children, QLF was used as a noninvasive adjunctive imaging method to reduce repeated radiographic exposure; however, QLF indicates plaque-associated fluorescence rather than clinically observed remineralization. Instead of clinical radiography, we conducted an in vitro study combining µ-CT and QLF, which suggested an association between QLF-area changes and thresholded enamel mineral volume under controlled conditions. Although residual plaque-associated deposits on extracted teeth may have been partially altered during extraction, transport, storage, and rinsing, the same procedures were applied to all specimens before and after treatment. Therefore, the observed QLF changes represent standardized changes in residual plaque-associated fluorescence under controlled experimental conditions rather than a biofilm culture model. Accordingly, the in vitro findings should be interpreted as changes in residual plaque-associated fluorescence rather than quantitative measurements of viable bacterial biomass. In the in vitro study, we did not distinguish ICDAS scores for extracted teeth, nor did we define whether caries was active or arrested. Besides, this clinical study provided ICDAS scores, but they are insufficient to determine whether the caries was active or arrested. Notably, ICDAS remained or decreased after all interventions, underscoring the importance of oral hygiene instruction.

From a statistical perspective, ICDAS scores were initially considered as potential covariates in the multivariable adjustment of the clinical QLF data. However, preliminary model evaluation showed that ICDAS scores did not meaningfully contribute to the outcome (*p* > 0.8), while their inclusion further reduced the model efficiency given the limited sample size. Therefore, the final ANCOVA model was restricted to baseline QLF value, age, and sex to provide a more parsimonious and interpretable adjustment. In addition, although the observed post-hoc power estimates appeared high, these values should be interpreted with caution because of the small sample size and exploratory nature of the clinical study. Accordingly, they should not be regarded as confirmatory evidence of adequate statistical power, but rather as preliminary estimates to guide sample size planning for future adequately powered trials.

Recent QLF literature supports fluorescence-based imaging as an adjunctive method for detecting and assessing caries-related fluorescence changes [17]. Red fluorescence has been associated with cariogenic biofilm properties in vitro [38]. Nevertheless, QLF red fluorescence may be influenced by plaque maturity, stains, image acquisition conditions, and ROI selection. In the present exploratory trial, QLF imaging showed reduced red fluorescence–covered areas after two months of calcium spray use, particularly in Formulation 1 (Fig. 5B–C), whereas minimal change was observed in the placebo group. Calcium ions may support enamel remineralization by contributing to hydroxyapatite formation and stabilization [39, 40] and by strengthening the salivary pellicle against acid challenge [41]. However, the mechanisms underlying the reduction in plaque-associated red fluorescence are likely multifactorial and were not directly examined in this study. Therefore, these findings should be interpreted as changes in plaque-associated fluorescence, together with increased salivary calcium and exploratory microbial trends, rather than direct evidence of clinical enamel remineralization, long-term caries prevention, or microbial causality.

This study has several limitations. First, the clinical trial included a relatively small sample size and should be considered exploratory. Because the participants ranged from 3 to 13 years of age, age-related differences in manual dexterity, oral hygiene behavior, and caregiver assistance may also have contributed to variability in QLF outcomes. Second, although all participants received standardized toothbrushing instructions and were provided with the same fluoride-free toothpaste, oral hygiene status was not formally assessed using clinical indices such as the Plaque Index (PI) or Gingival Index (GI). Therefore, changes in plaque-associated fluorescence could not be directly correlated with changes in oral hygiene or gingival health. Third, salivary pH was not measured, and its potential influence on demineralization, remineralization, and microbial activity could not be evaluated. Fourth, dietary habits and post-application eating or drinking behaviors were not standardized or monitored, which may have affected plaque accumulation, salivary composition, and QLF outcomes. Accordingly, the observed changes in fluorescence-detectable plaque-associated area should be interpreted cautiously and cannot be attributed exclusively to the tested formulations. Future studies with larger sample sizes, comprehensive clinical indices, salivary pH measurements, and controlled dietary assessments are warranted. In addition, lesion activity, lesion hardness, and caries arrest were not evaluated; therefore, the present findings should not be interpreted as direct evidence of clinical caries reversal or enamel remineralization.

## Conclusions

In this exploratory study, both calcium-containing oral sprays were associated with greater reductions in the primary clinical outcome, fluorescence-detectable plaque-associated area, compared with placebo. No statistically significant difference in the primary clinical outcome was observed between the calcium-containing formulation and calcium-plus-fluoride formulation. In the separate in vitro experiment, threshold-segmented enamel mineralized volume showed numerically higher post-treatment values in the artificial saliva and calcium-containing solution groups, and ΔMV was significantly negatively correlated with ΔQLF-detected fluorescent area. Because the in vitro and clinical components addressed different research questions, these findings should be interpreted independently and as hypothesis-generating. Larger, adequately powered randomized clinical trials are required.

## Supplementary Information

Supplementary Material 1.

Supplementary Material 2.

## Data Availability

The 16 S rRNA sequencing data generated in this study have been deposited in the NCBI BioProject database under accession number PRJNA1404812.

## Abbreviations

QLF: Quantitative Light-induced Fluorescence
MV: Threshold-segmented enamel mineralized volume
QLF-areas: QLF-covered areas
SEM: Scanning electron microscope
µ-CT: Micro-computed tomography
RCT: Randomized clinical trial

## References

[1] Okada M, Soda Y, Hayashi F, Doi T, Suzuki J, Miura K, et al. Longitudinal study of dental caries incidence associated with Streptococcus mutans and Streptococcus sobrinus in pre-school children. J Med Microbiol. 2005;54(7):661–5. 10.1099/jmm.0.46069-0 15947431

[2] Zero DT. Dental caries process. Dent Clin North Am. 1999;43(4):635–64. 10553248

[3] Kidd EAM, Fejerskov O. What Constitutes Dental Caries? Histopathology of Carious Enamel and Dentin Related to the Action of Cariogenic Biofilms. J Dent Res. 2004;83(Suppl 1):35–8. 10.1177/154405910408301s07 15286119

[4] Featherstone JDB. Remineralization, the Natural Caries Repair Process—The Need for New Approaches. Adv Dent Res. 2009;21(1):4–7. 10.1177/0895937409335590 19717404

[5] Abou Neel EA, Aljabo A, Strange A, Ibrahim S, Coathup M, Young AM, et al. Demineralization-remineralization dynamics in teeth and bone. Int J Nanomed. 2016;11:4743–63. 10.2147/IJN.S107624 PMC5034904 27695330

[6] Amaechi BT, van Loveren C. Fluorides and non-fluoride remineralization systems. Monogr Oral Sci. 2013;23:15–26. 10.1159/000350458 23817057

[7] Whelton HP, Spencer AJ, Do LG, Rugg-Gunn AJ. Fluoride Revolution and Dental Caries: Evolution of Policies for Global Use. J Dent Res. 2019;98(8):837–46. 10.1177/0022034519843495 31282846

[8] Aoun A, Darwiche F, Al Hayek S, Doumit J. The Fluoride Debate: The Pros and Cons of Fluoridation. Prev Nutr Food Sci. 2018;23(3):171–80. 10.3746/pnf.2018.23.3.171 PMC6195894 30386744

[9] Meyer F, Amaechi BT, Fabritius HO, Enax J. Overview of Calcium Phosphates used in Biomimetic Oral Care. Open Dent J. 2018;12:406–23. 10.2174/1874210601812010406 PMC5997847 29988215

[10] Singer L, Fouda A, Bourauel C. Biomimetic approaches and materials in restorative and regenerative dentistry: review article. BMC Oral Health. 2023;23(1):105. 10.1186/s12903-023-02808-3 PMC9936671 36797710

[11] Chen J, Zhang Y, Yin I, Yu OY, Chan A, Chu C-H. Preventing Dental Caries with Calcium-Based Materials: A Concise Review. Inorganics. 2024;12:253.

[12] Cai F, Shen P, Walker GD, Reynolds C, Yuan Y, Reynolds EC. Remineralization of enamel subsurface lesions by chewing gum with added calcium. J Dent. 2009;37(10):763–8. 10.1016/j.jdent.2009.06.003 19596505

[13] Zhou C, Zhang D, Bai Y, Li S. Casein phosphopeptide–amorphous calcium phosphate remineralization of primary teeth early enamel lesions. J Dent. 2014;42(1):21–9. 10.1016/j.jdent.2013.11.005 24269831

[14] Amaechi BT, AbdulAzees PA, Alshareif DO, Shehata MA, Lima PPCS, Abdollahi A, et al. Comparative efficacy of a hydroxyapatite and a fluoride toothpaste for prevention and remineralization of dental caries in children. BDJ Open. 2019;5(1):18. 10.1038/s41405-019-0026-8 PMC6901576 31839988

[15] Chen KJ, Gao SS, Duangthip D, Lo ECM, Chu CH. Randomized Clinical Trial on Sodium Fluoride with Tricalcium Phosphate. J Dent Res. 2021;100(1):66–73. 10.1177/0022034520952031 PMC7754823 32866050

[16] Madawana AM, Baharin F, Taib H, Mamat N, Nawi MAA, Rahman FSA. Effect of Mouth Spray Enriched With Salivary Enzymes on the Oral Health Status among Young Children-A Randomized Controlled Trial. Ann Abbasi Shaheed Hosp Karachi Med Dent Coll. 2025;30(2):87–94.

[17] Bittar A, Alkan E, Kanar Ö, Korkut B, Tağtekin D. Comparison of quantitative light-induced fluorescence and near-infrared intraoral scanner imaging for proximal caries across predefined ICCMS radiographic thresholds: a diagnostic accuracy study. BMC Oral Health. 2026;26(1):1365. 10.1186/s12903-026-08559-1 PMC13429018 42169111

[18] Ionta FQ, Mendonça FL, de Oliveira GC, de Alencar CR, Honório HM, Magalhães AC, et al. In vitro assessment of artificial saliva formulations on initial enamel erosion remineralization. J Dent. 2014;42(2):175–9. 10.1016/j.jdent.2013.11.009 24269764

[19] van der Veen MH, Volgenant CMC, Keijser B, ten Cate JM, Crielaard W. Dynamics of red fluorescent dental plaque during experimental gingivitis—A cohort study. J Dent. 2016;48:71–6. 10.1016/j.jdent.2016.02.010 26921667

[20] Neves AA, Coutinho E, Vivan Cardoso M, Jaecques SV, Van Meerbeek B. Micro-CT based quantitative evaluation of caries excavation. Dent Mater. 2010;26(6):579–88. 10.1016/j.dental.2010.01.012 20347481

[21] Crescente CL, de Sousa ET, Lima-Holanda AT, Steiner-Oliveira C, Nobre-dos-Santos M. Biofilm accumulation and sucrose rinse modulate calcium and fluoride bioavailability in the saliva of children with early childhood caries. Sci Rep. 2022;12(1):10283. 10.1038/s41598-022-14583-2 PMC9206641 35717506

[22] Sams CM, Dietsche EW, Swenson DW, DuPont GJ, Ayyala RS. Pediatric Panoramic Radiography: Techniques, Artifacts, and Interpretation. Radiographics. 2021;41(2):595–608. 10.1148/rg.2021200112 33513075

[23] Pontes LRA, Novaes TF, Lara JS, Moro BLP, Gimenez T, Raggio DP, et al. Impact of the radiographic method on treatment decisions related to dental caries in primary molars: a before–after study. Clin Oral Investig. 2019;23(11):4075–81. 10.1007/s00784-019-02844-w 30778687

[24] Abdelaziz M. Detection, Diagnosis, and Monitoring of Early Caries: The Future of Individualized Dental Care. Diagnostics (Basel). 2023;13(24):3649. 10.3390/diagnostics13243649 PMC10742918 38132233

[25] Coulthwaite L, Pretty IA, Smith PW, Higham SM, Verran J. The Microbiological Origin of Fluorescence Observed in Plaque on Dentures during QLF Analysis. Caries Res. 2006;40(2):112–6. 10.1159/000091056 16508267

[26] Lee E-S, Kang S-M, Ko H-Y, Kwon H-K, Kim B-I. Association between the cariogenicity of a dental microcosm biofilm and its red fluorescence detected by Quantitative Light-induced Fluorescence-Digital (QLF-D). J Dent. 2013;41(12):1264–70. 10.1016/j.jdent.2013.08.021 24012520

[27] Cengiz IF, Oliveira JM, Reis RL. Micro-CT – a digital 3D microstructural voyage into scaffolds: a systematic review of the reported methods and results. Biomater Res. 2018;22(1):26. 10.1186/s40824-018-0136-8 PMC6158835 30275969

[28] Jiang W, Ling Z, Lin X, Chen Y, Zhang J, Yu J, et al. Pyrosequencing Analysis of Oral Microbiota Shifting in Various Caries States in Childhood. Microb Ecol. 2014;67(4):962–9. 10.1007/s00248-014-0372-y 24504329

[29] Teng F, Yang F, Huang S, Bo C, Xu ZZ, Amir A, et al. Prediction of Early Childhood Caries via Spatial-Temporal Variations of Oral Microbiota. Cell Host Microbe. 2015;18(3):296–306. 10.1016/j.chom.2015.08.005 26355216

[30] Takahashi N, Nyvad B. The role of bacteria in the caries process: ecological perspectives. J Dent Res. 2011;90(3):294–303. 10.1177/0022034510379602 20924061

[31] Dipalma G, Inchingolo AD, Inchingolo F, Charitos IA, Di Cosola M, Cazzolla AP. Focus on the cariogenic process: microbial and biochemical interactions with teeth and oral environment. J Biol Regul Homeost Agents. 2021;35(2):429–40. 10.23812/20-747-A 33904270

[32] Shen P, Bagheri R, Walker G, Yuan Y, Stanton D, Reynolds C, et al. Effect of calcium phosphate addition to fluoride containing dental varnishes on enamel demineralization. Aust Dent J. 2016;61(3):357–65. 10.1111/adj.12385 26541509

[33] Vicente A, Ortiz-Ruiz AJ, González-Paz BM, Martínez-Beneyto Y, Bravo-González LA. Effectiveness of a toothpaste and a serum containing calcium silicate on protecting the enamel after interproximal reduction against demineralization. Sci Rep. 2021;11(1):834. 10.1038/s41598-020-80844-7 PMC7804454 33437013

[34] Shen P, Walker GD, Yuan Y, Reynolds C, Stanton DP, Fernando JR, et al. Importance of bioavailable calcium in fluoride dentifrices for enamel remineralization. J Dent. 2018;78:59–64. 10.1016/j.jdent.2018.08.005 30099066

[35] Menon LU, Varma RB, Kumaran P, Xavier AM, Govinda BS, Kumar JS. Efficacy of a Calcium Sucrose Phosphate Based Toothpaste in Elevating the Level of Calcium, Phosphate Ions in Saliva and Reducing Plaque: A Clinical Trial. Contemp Clin Dent. 2018;9(2):151–7. 10.4103/ccd.ccd_562_17 PMC5968674 29875552

[36] Lynch RM. Calcium glycerophosphate and caries: a review of the literature. Int Dent J. 2004;54(5 Suppl 1):310–4. 10.1111/j.1875-595x.2004.tb00004.x 15509082

[37] Cavazana TP, Hosida TY, Sampaio C, de Morais LA, Monteiro DR, Pessan JP, et al. Calcium glycerophosphate and fluoride affect the pH and inorganic composition of dual-species biofilms of Streptococcus mutans and Candida albicans. J Dent. 2021;115:103844. 10.1016/j.jdent.2021.103844 34637893

[38] Akifusa S, Isobe A, Kibata K, Oyama A, Oyama H, Ariyoshi W, et al. Comparison of dental plaque reduction after use of electric toothbrushes with and without QLF-D-applied plaque visualization: a 1-week randomized controlled trial. BMC Oral Health. 2020;20(1):4. 10.1186/s12903-019-0982-3 PMC6996165 32008572

[39] Dong H, Wang D, Deng H, Yin L, Wang X, Yang W, et al. Application of a calcium and phosphorus biomineralization strategy in tooth repair: a systematic review. J Mater Chem B. 2024;12(33):8033–47. 10.1039/d4tb00867g 39045831

[40] Cochrane NJ, Cai F, Huq NL, Burrow MF, Reynolds EC. New Approaches to Enhanced Remineralization of Tooth Enamel. J Dent Res. 2010;89(11):1187–97. 10.1177/0022034510376046 20739698

[41] Chawhuaveang DD, Yu OY, Yin IX, Lam WY-H, Mei ML, Chu C-H. Acquired salivary pellicle and oral diseases: A literature review. J Dent Sci. 2021;16(1):523–9. 10.1016/j.jds.2020.10.007 PMC7770358 33384841

