# Supplementary material for: Effects of calcium-containing solutions on fluorescence-detectable plaque area and enamel mineral volume: an in vitro and exploratory clinical study
Source: BMC Oral Health. 2026 Sep 15;26:1873. doi: 10.1186/s12903-026-09789-z (PMC13628852; doi:10.1186/s12903-026-09789-z)
Supplement: Supplementary file 1 [file 12903_2026_9789_MOESM1_ESM.pdf]

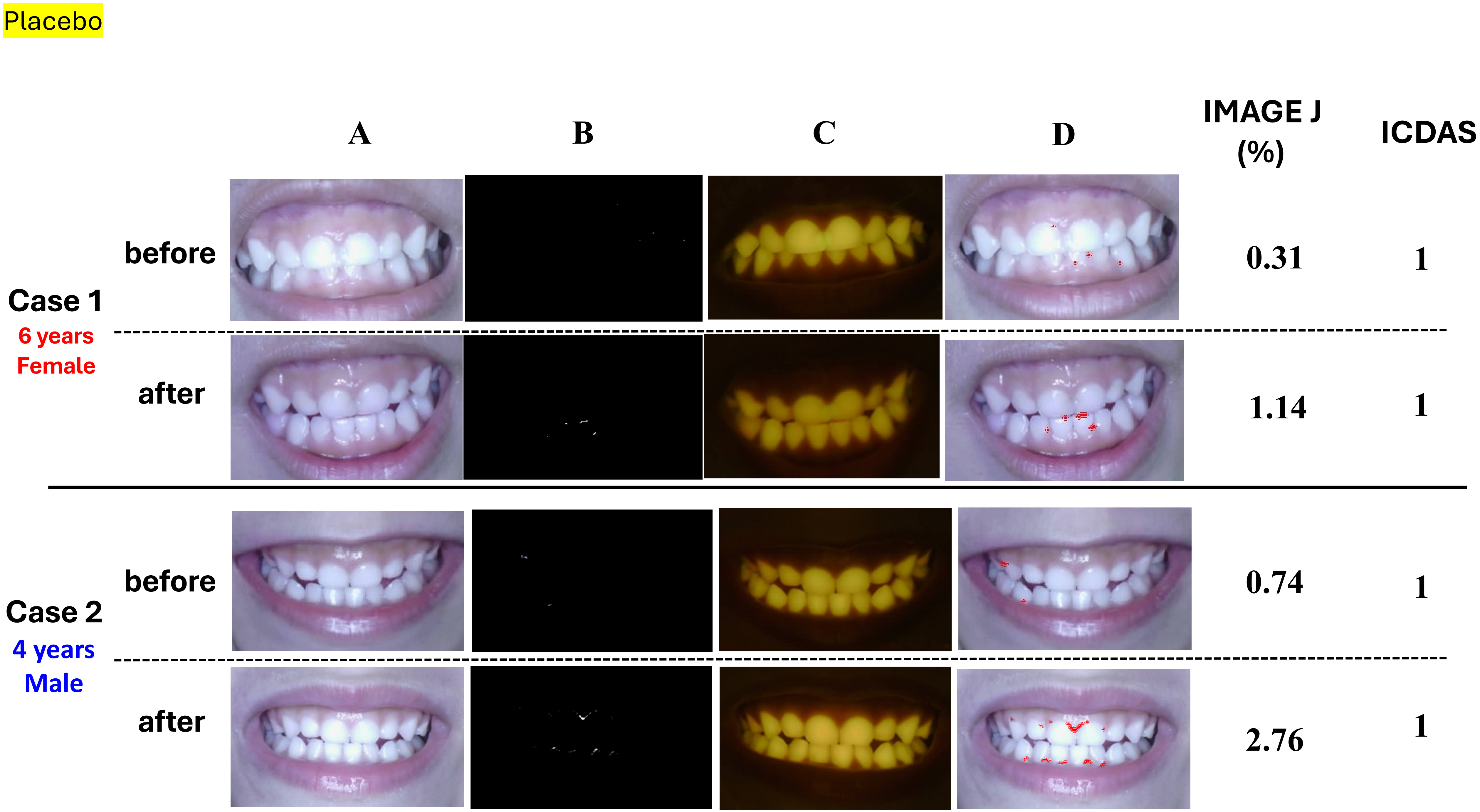
Placebo
A
B
C
D
IMAGE J (%)
ICDAS
Case 1
6 years
Female
before
0.31
1
after
1.14
1
Case 2
4 years
Male
before
0.74
1
after
2.76
1

Placebo

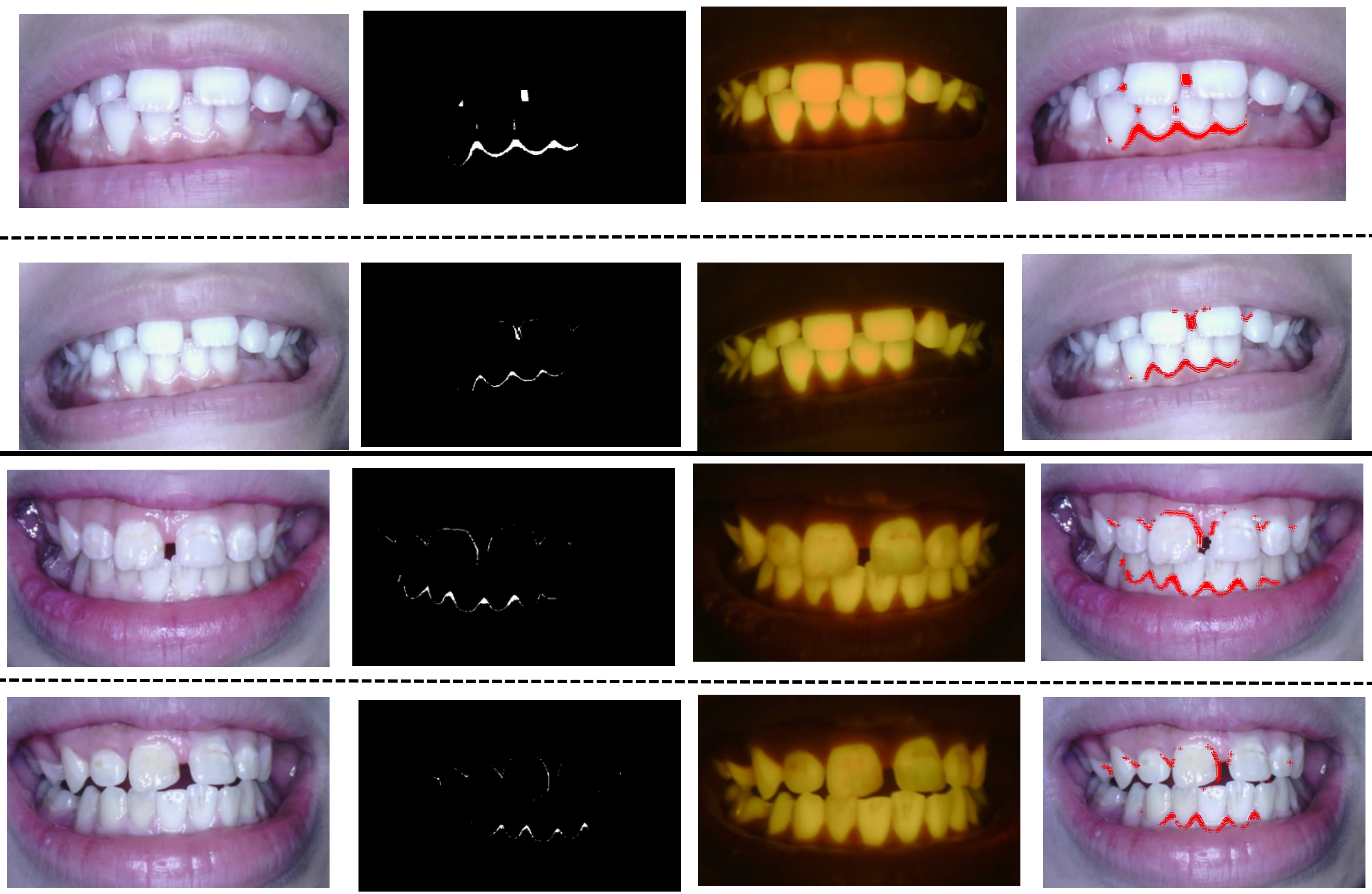

Case		A / B / C / D	IMAGE J (%)	ICDAS
Case 3<br>4 years<br>Female	before		5.00	1
	after		5.28	1
Case 4<br>12 years<br>Male	before		3.88	1
	after		3.50	1

Placebo

		A	B	C	D	IMAGE J (%)	ICDAS
**Case 5**<br>8 years<br>Male	before	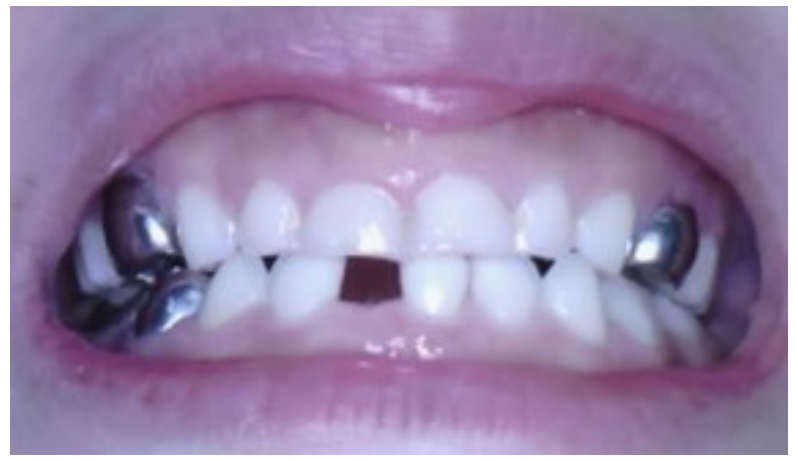	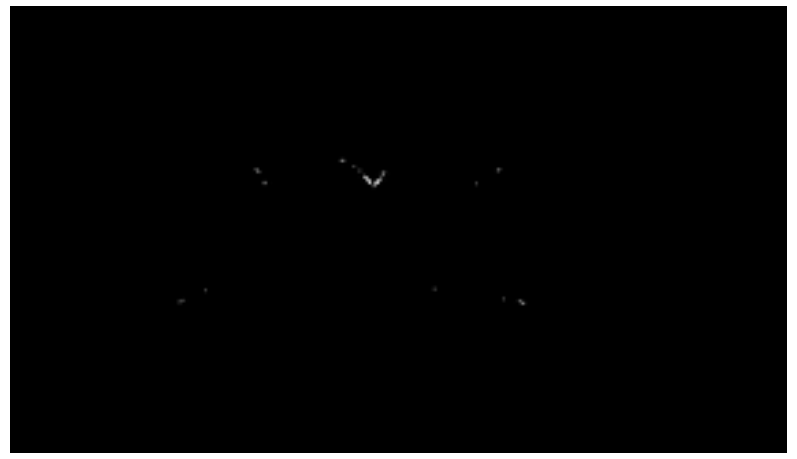	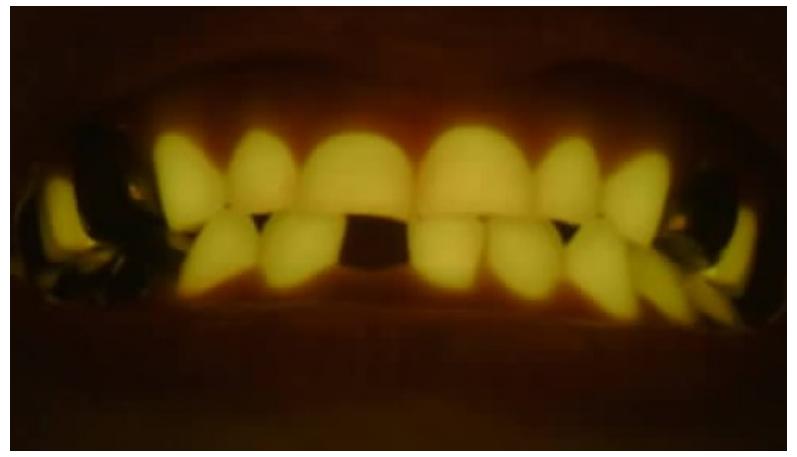	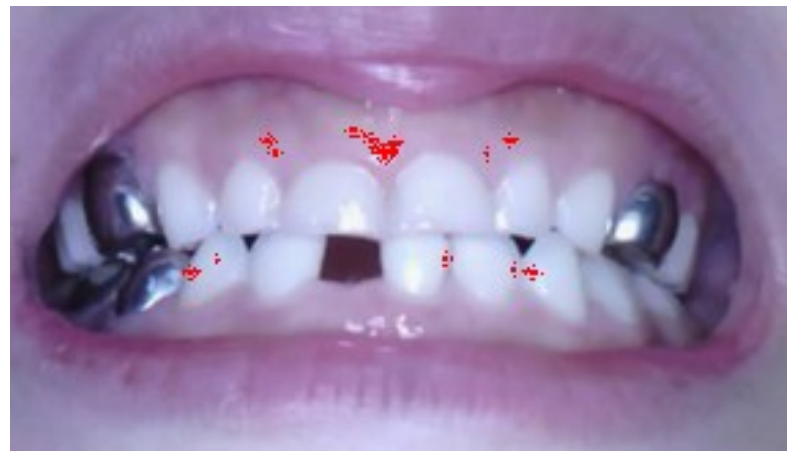	2.416	1
	after	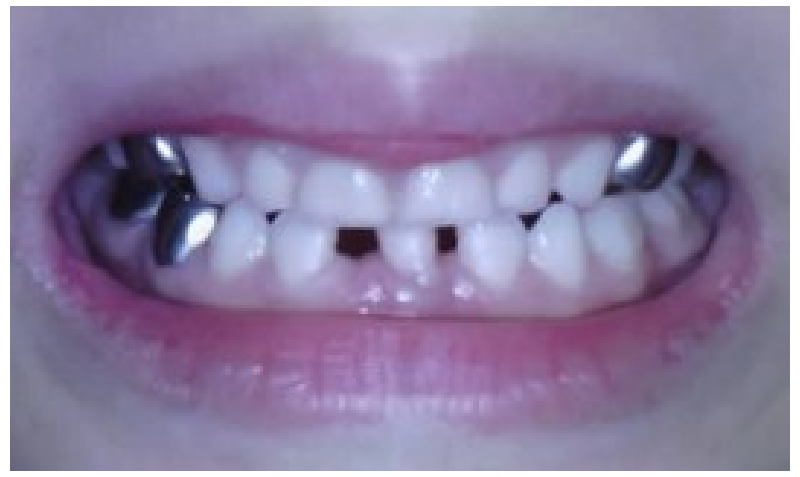	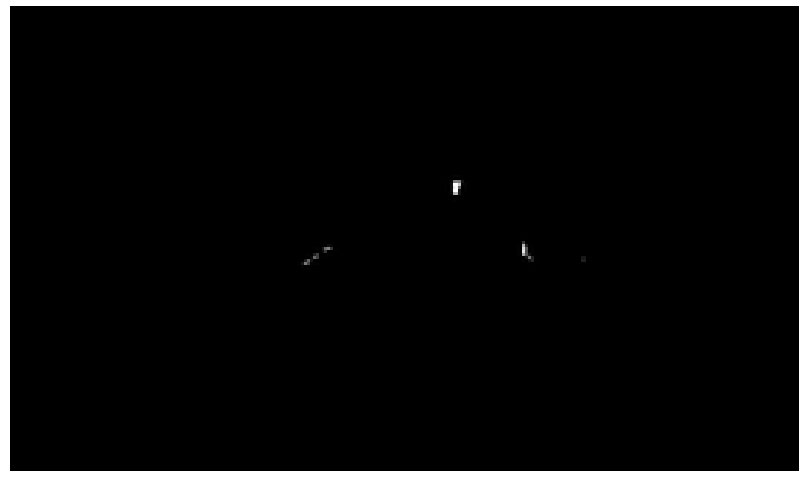	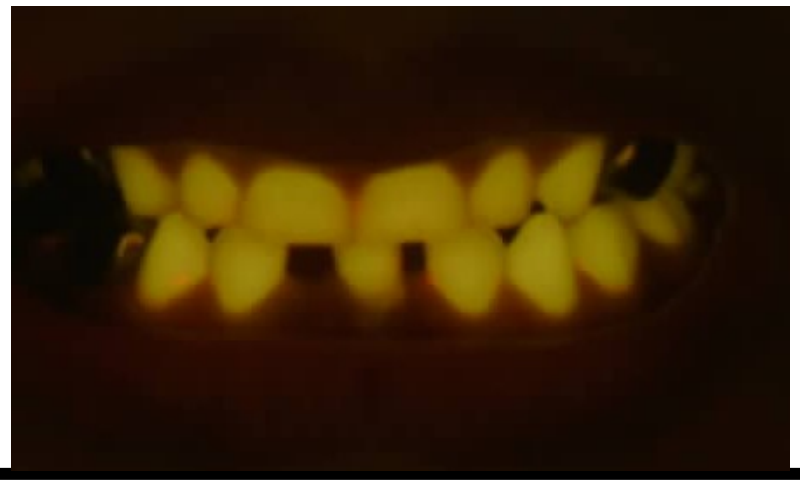	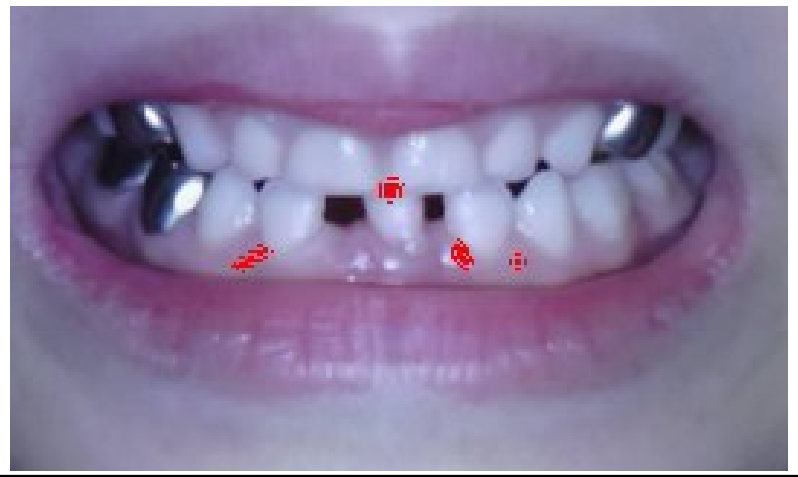	2.227	1

Formulation 1

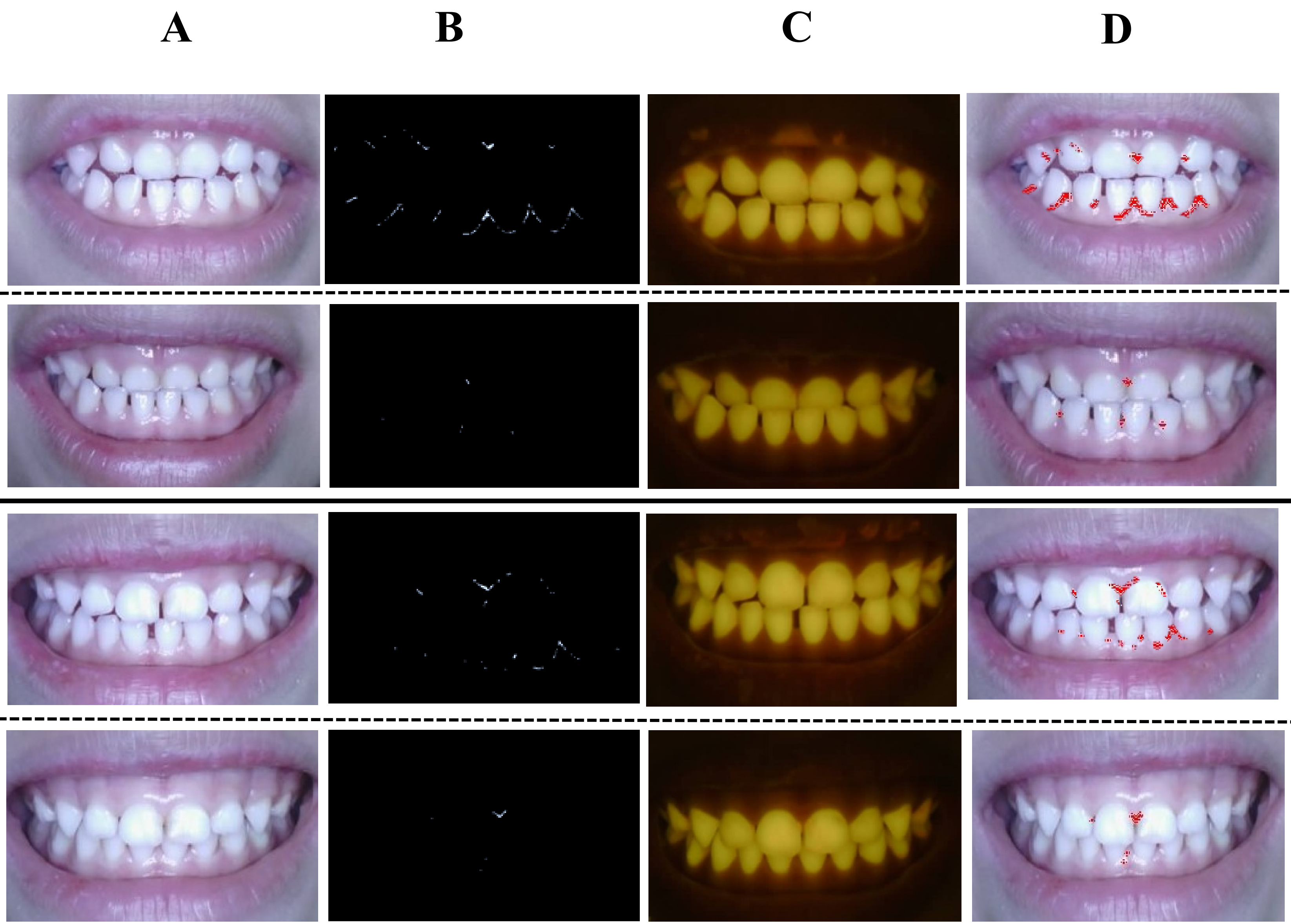

Case		IMAGE J (%)	ICDAS
Case 6 (6 years, Female)	before	5.490	2
	after	1.338	1
Case 7 (6 years, Female)	before	5.479	1、2
	after	2.757	1

Formulation 1

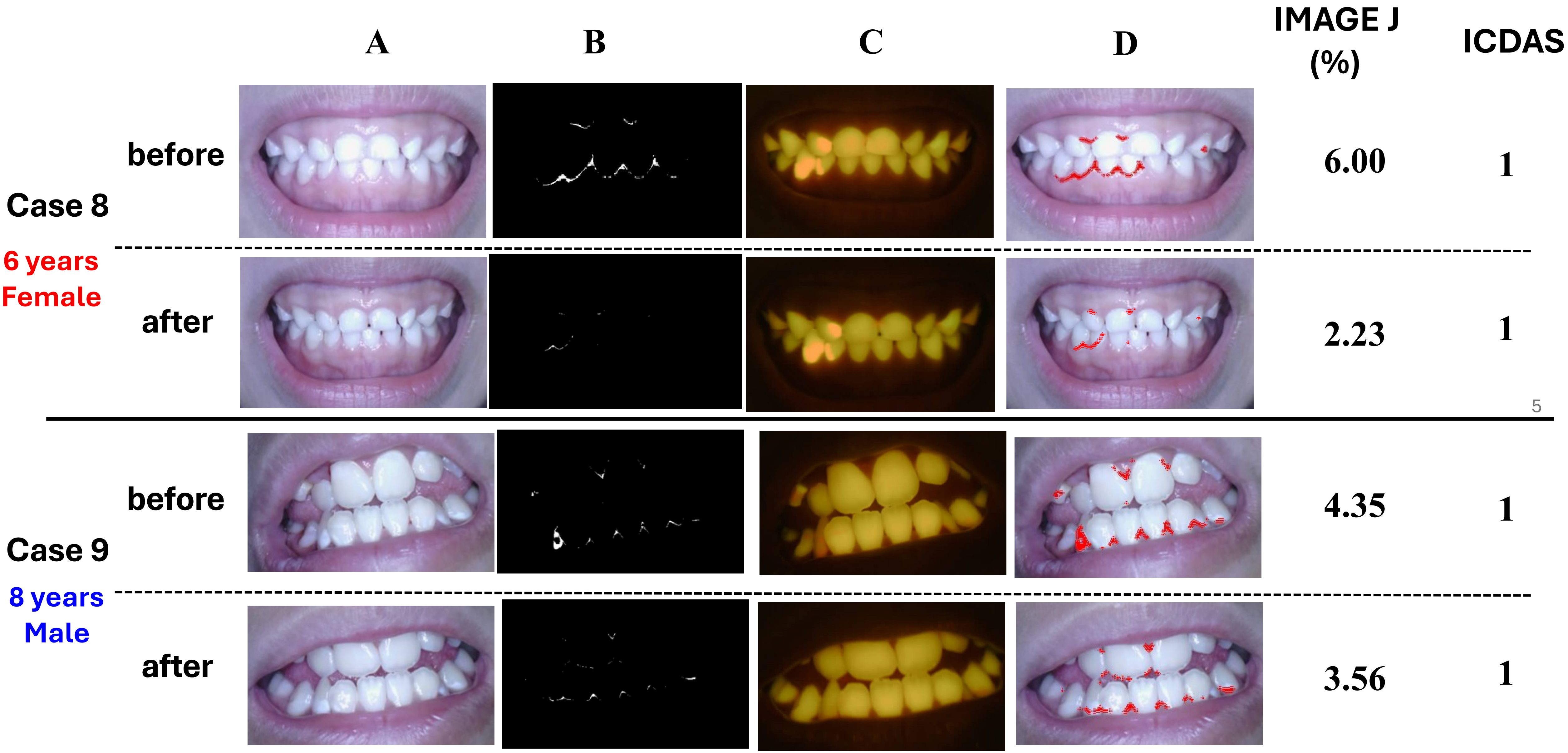

Formulation 1

		A	B	C	D	IMAGE J (%)	ICDAS
Case 10<br>8 years<br>Male	before	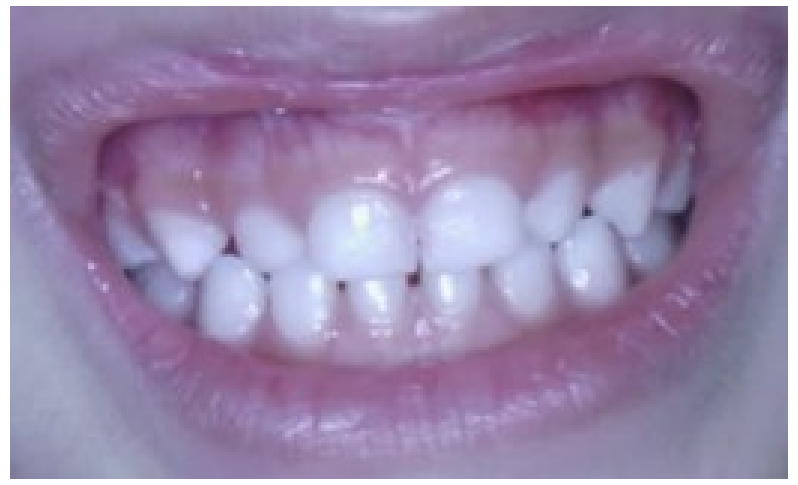	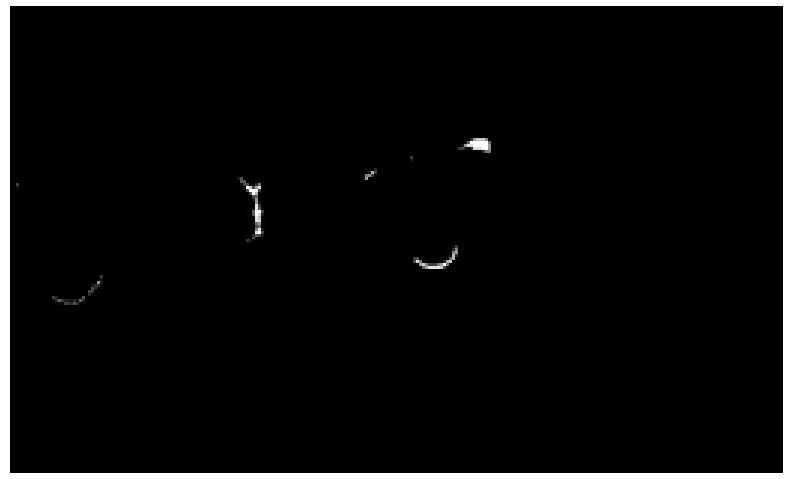	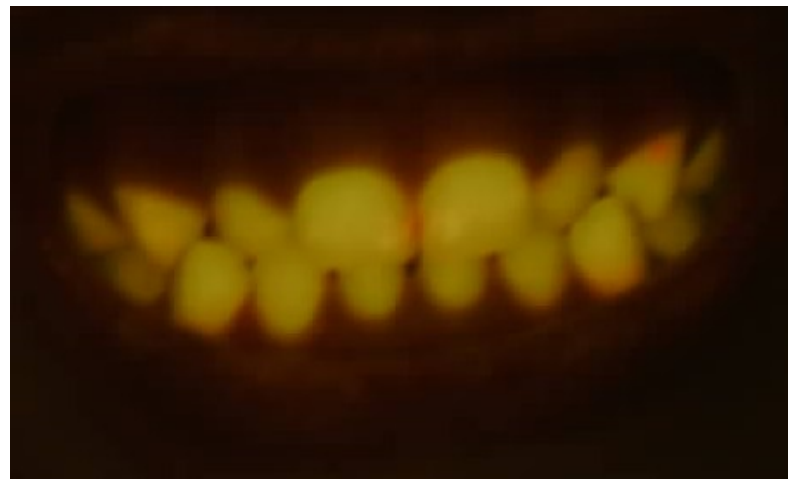	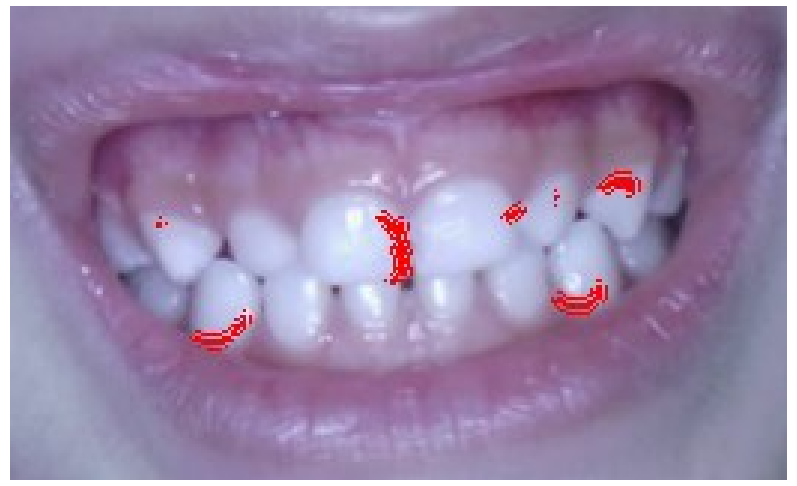	4.344	1
	after	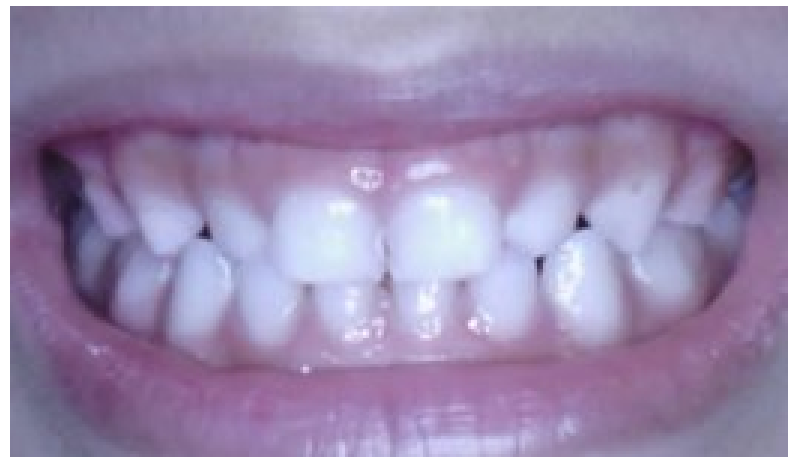	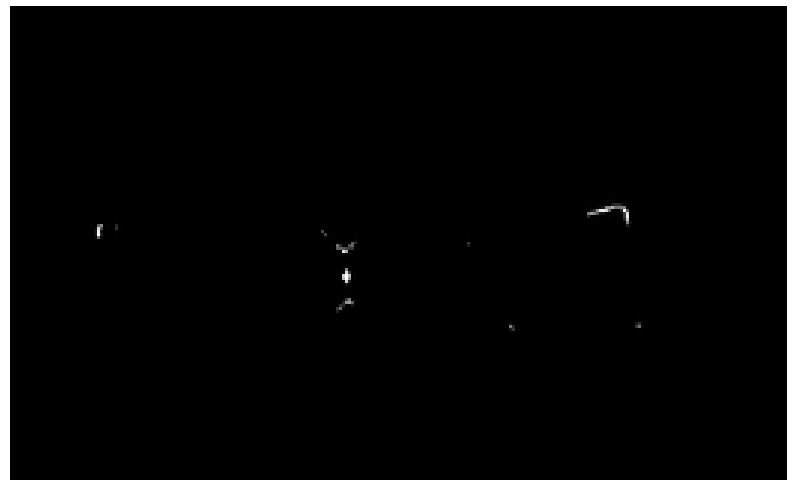	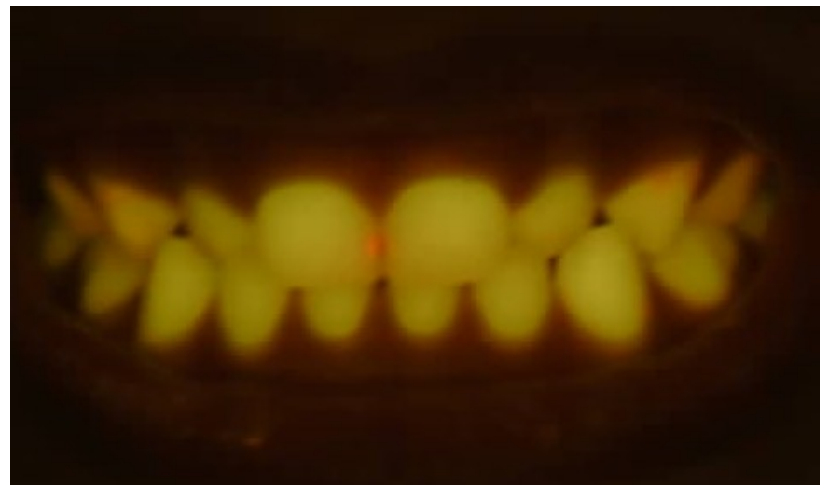	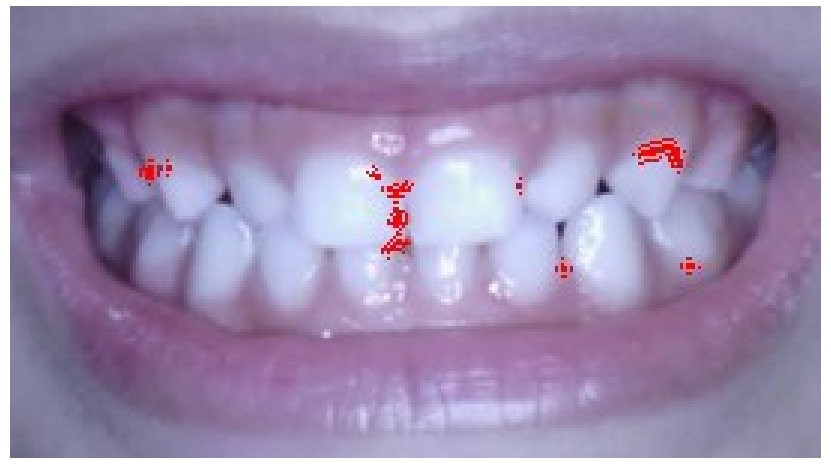	1.94	1

Formulation 2

Case		A	B	C	D	IMAGE J (%)	ICDAS
Case 11 4 years Male	before	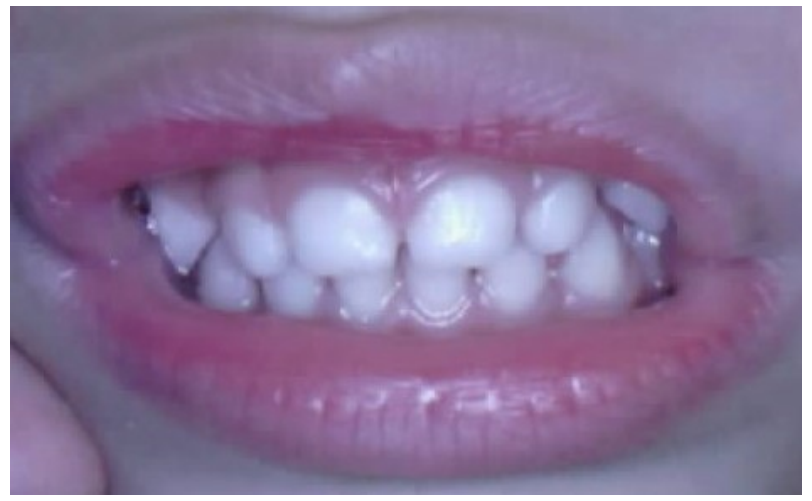	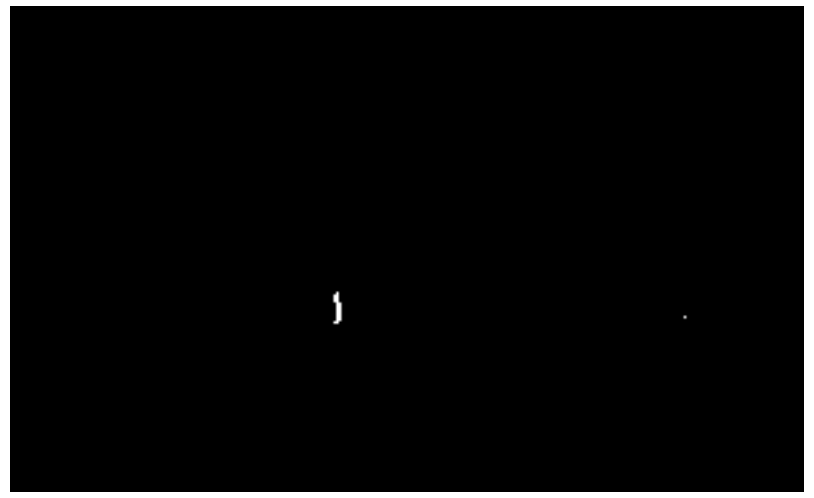	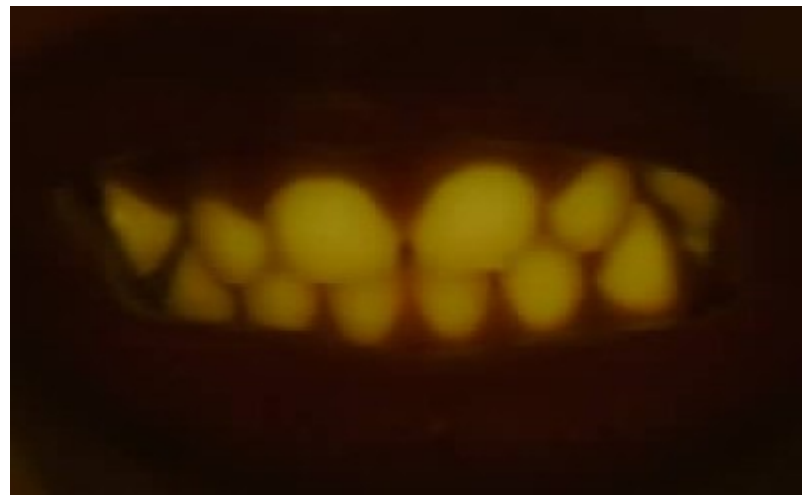	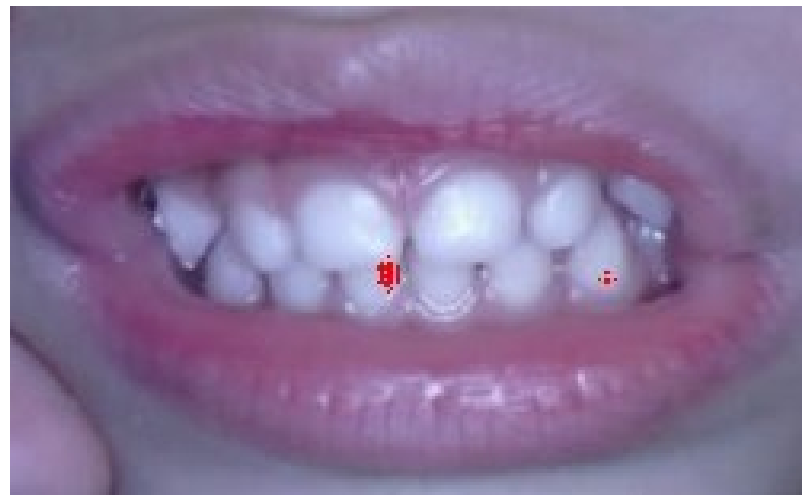	0.69	1
	after	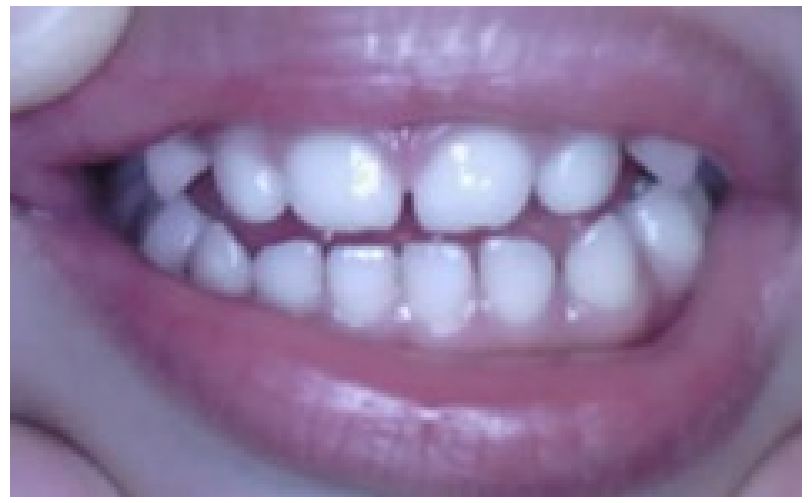	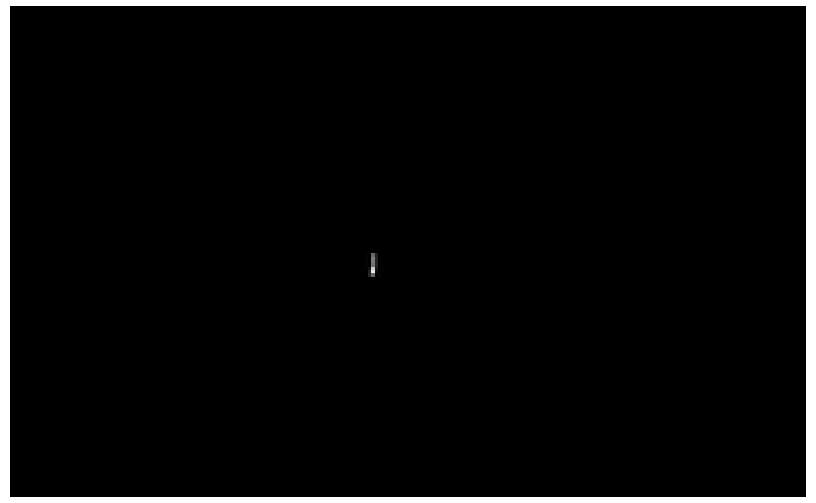	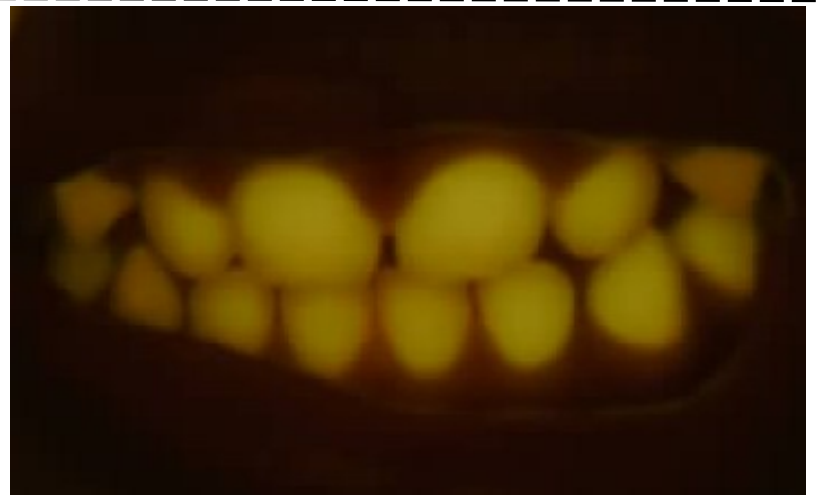	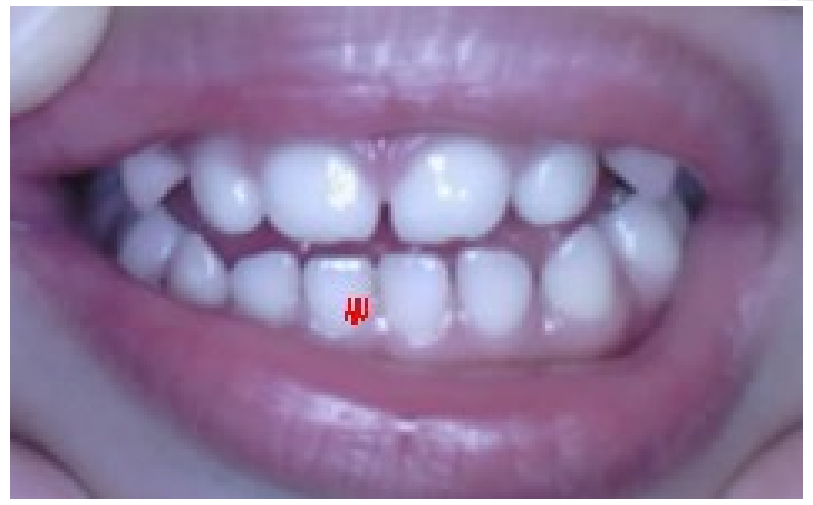	0.48	1
Case 12 13 years Male	before	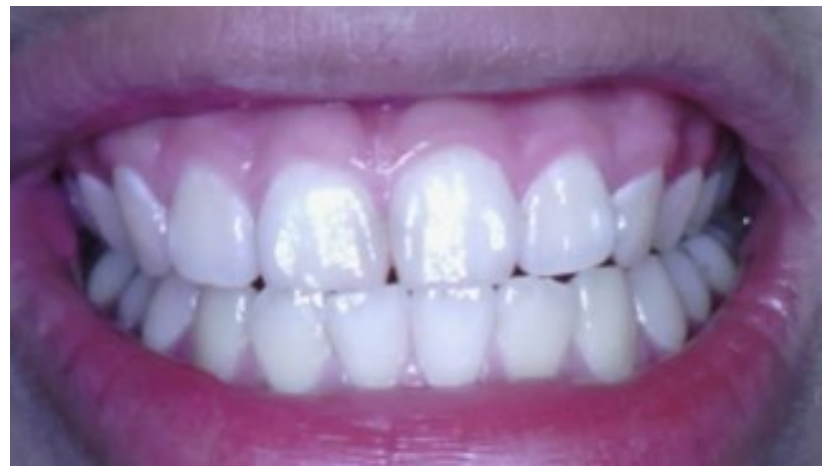	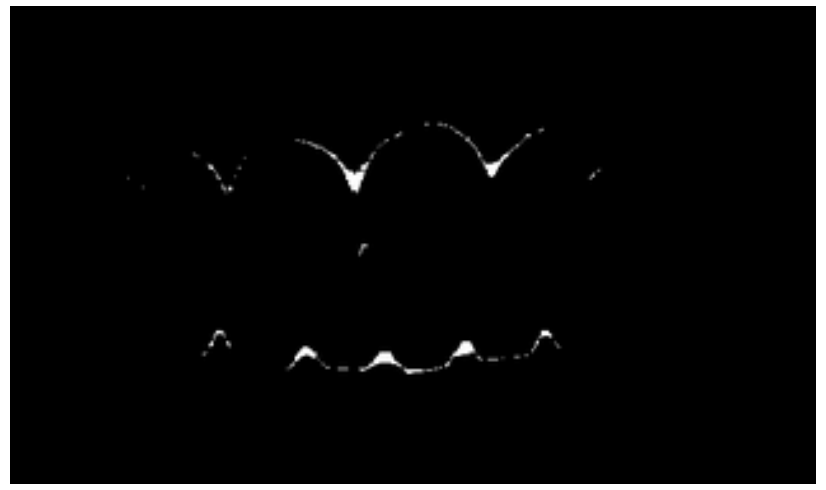	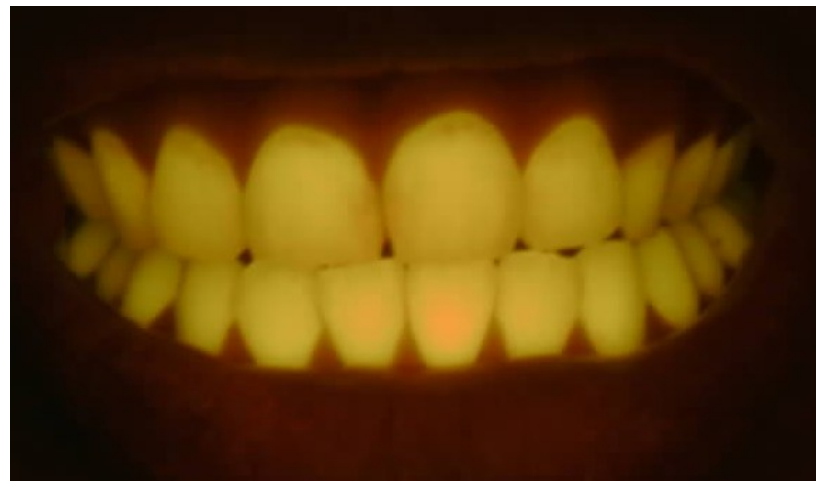	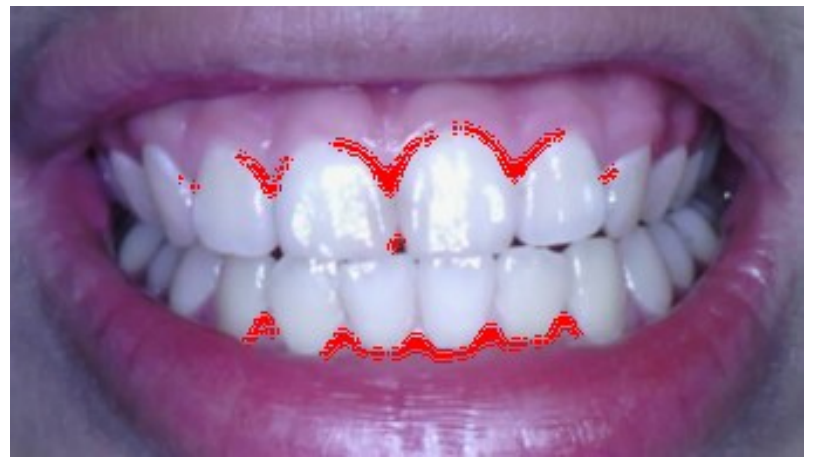	5.92	1
	after	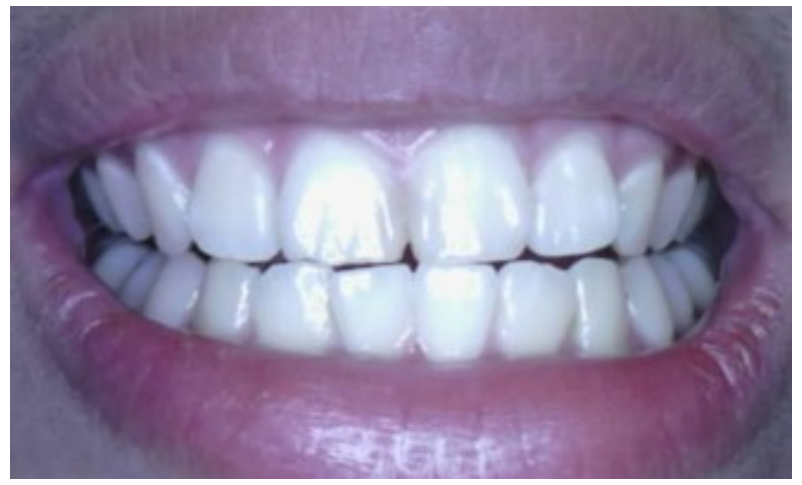	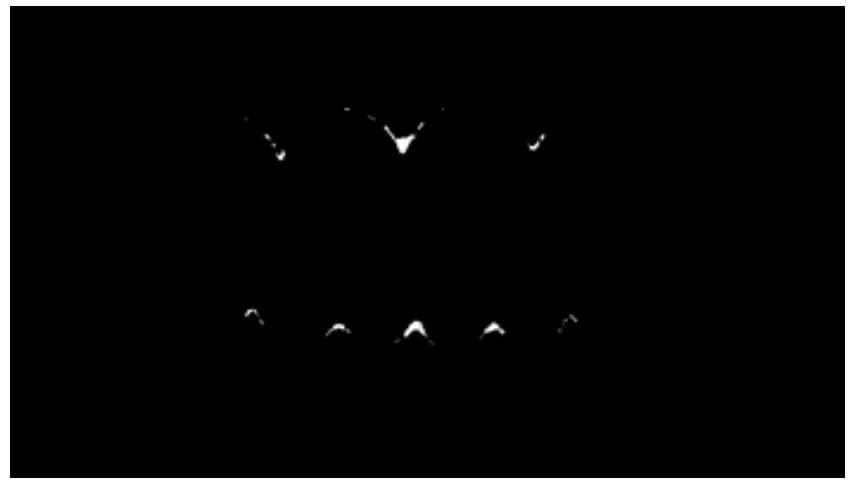	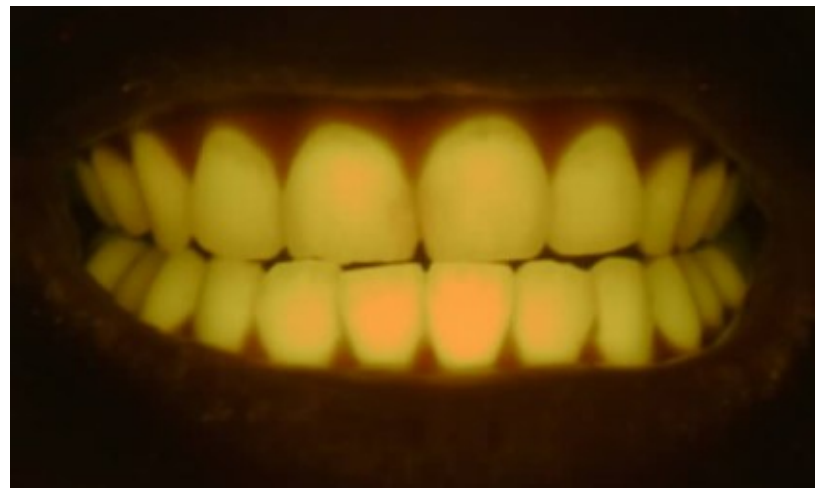	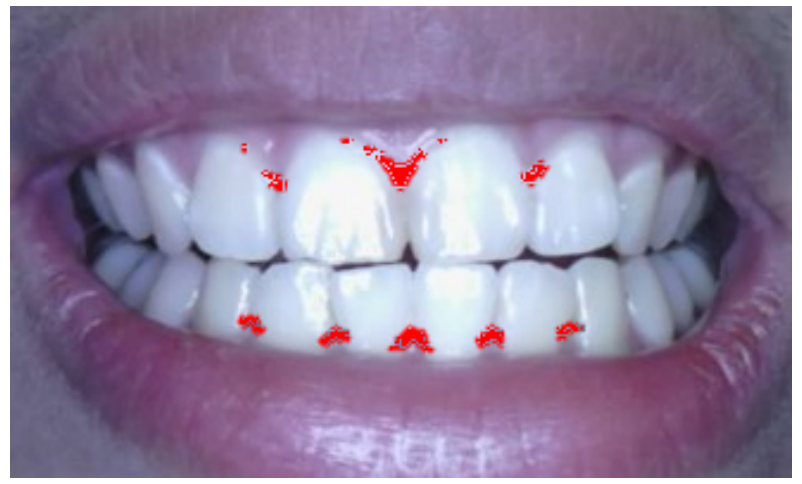	3.06	1

Formulation 2

		A B C D	IMAGE J (%)	ICDAS
Case 13<br>4 years<br>Female	before	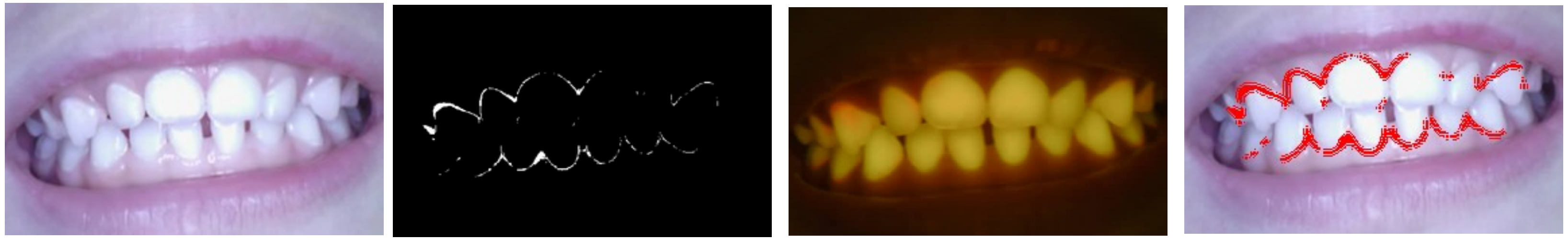	8.66	1
	after	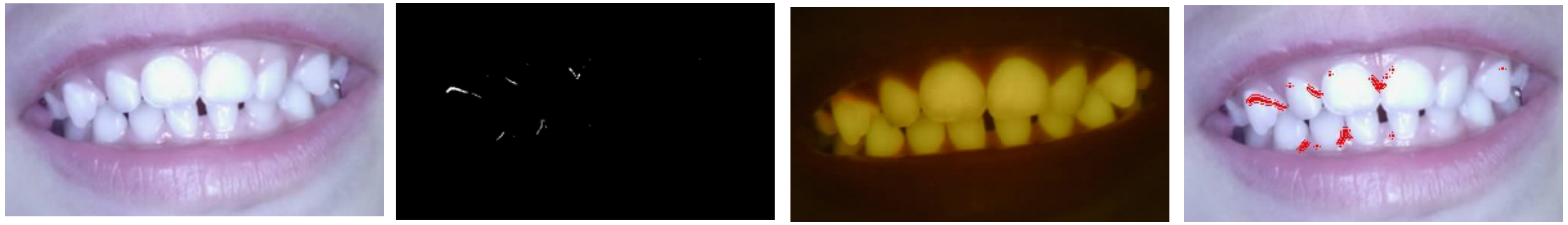	2.17	1
Case 14<br>3 years<br>Female	before	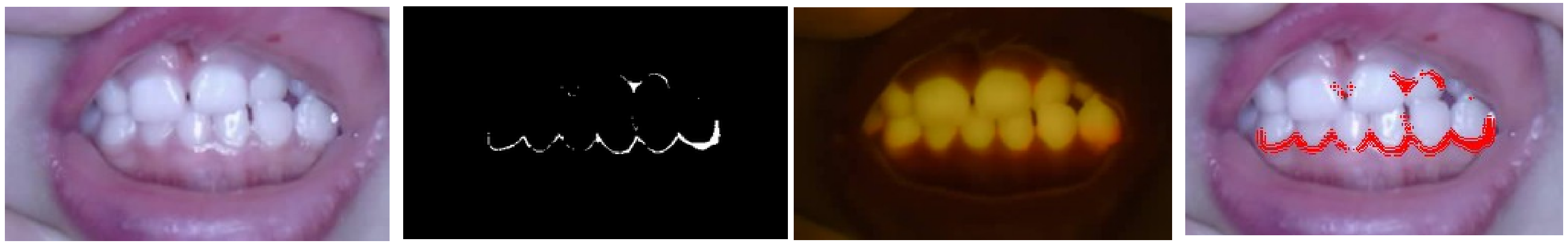	10.15	1
	after	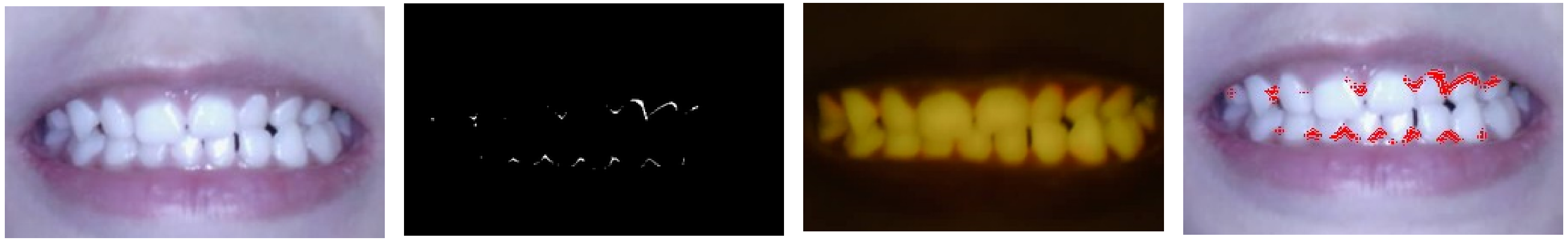	7.41	1

Formulation 2

		A	B	C	D	IMAGE J (%)	ICDAS
Case 15	before	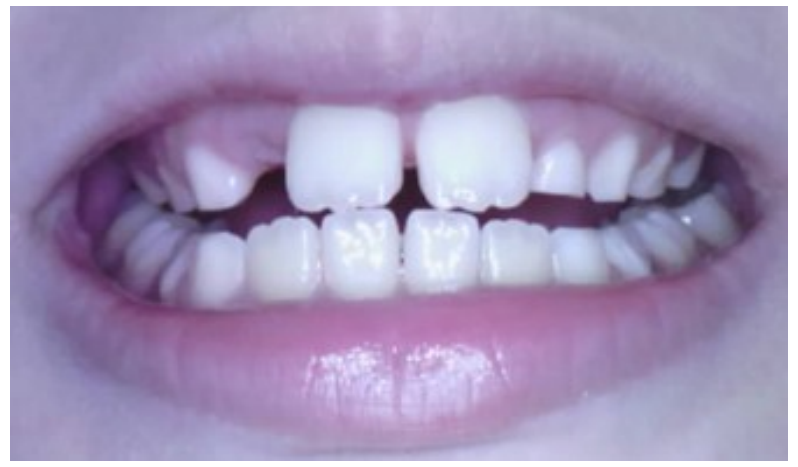	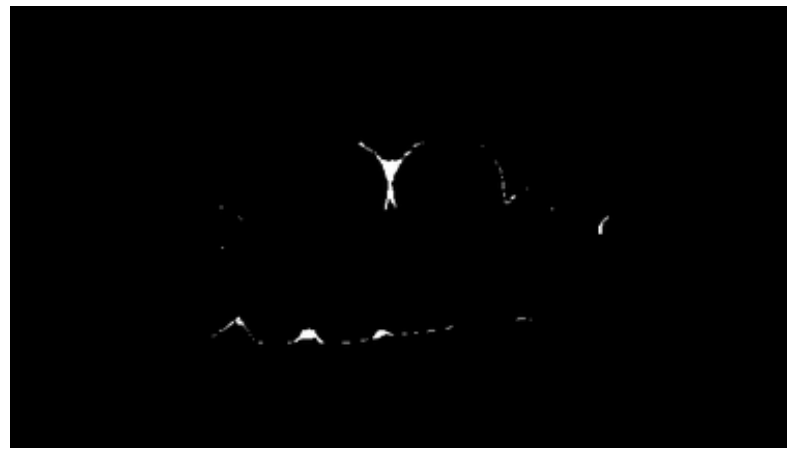	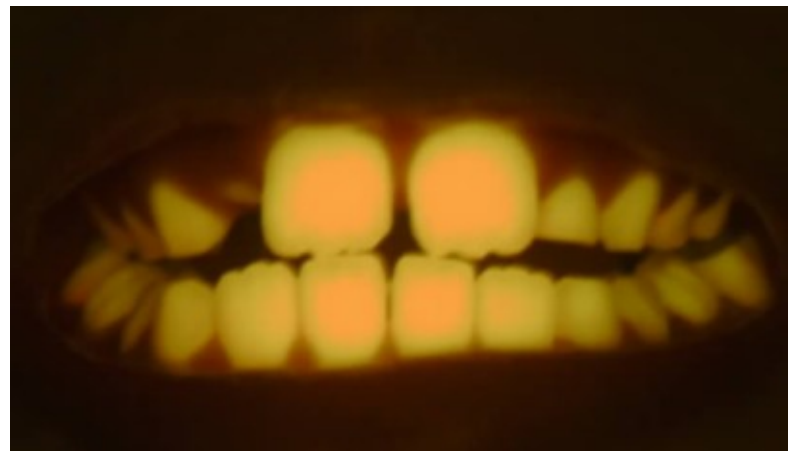	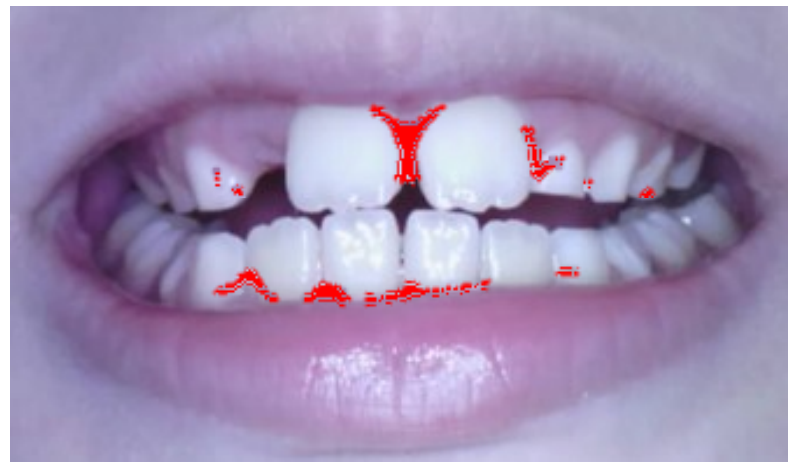	8.63	1
8 years Male	after	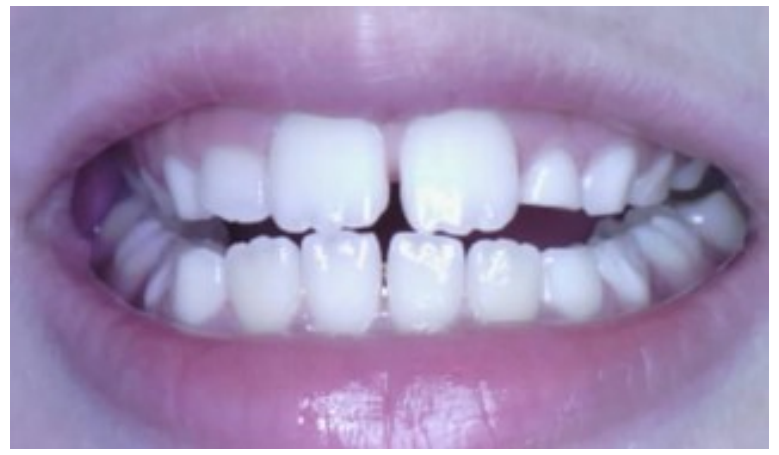	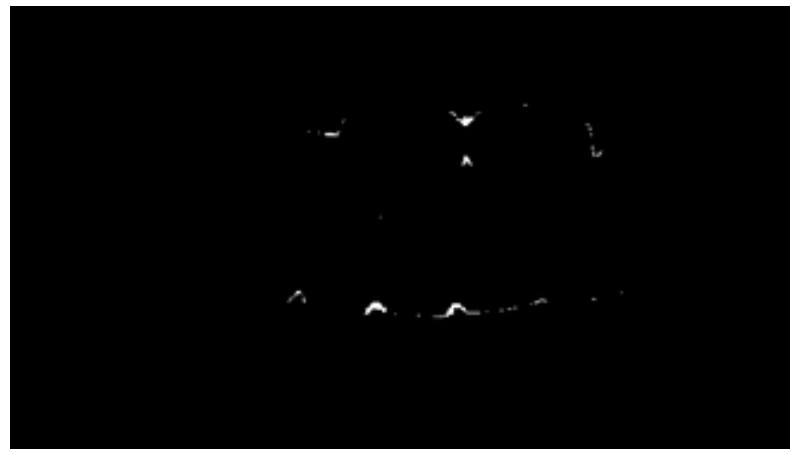	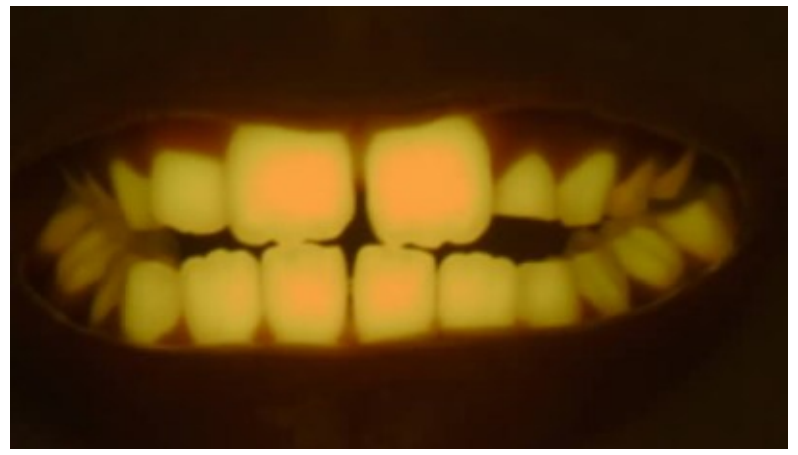	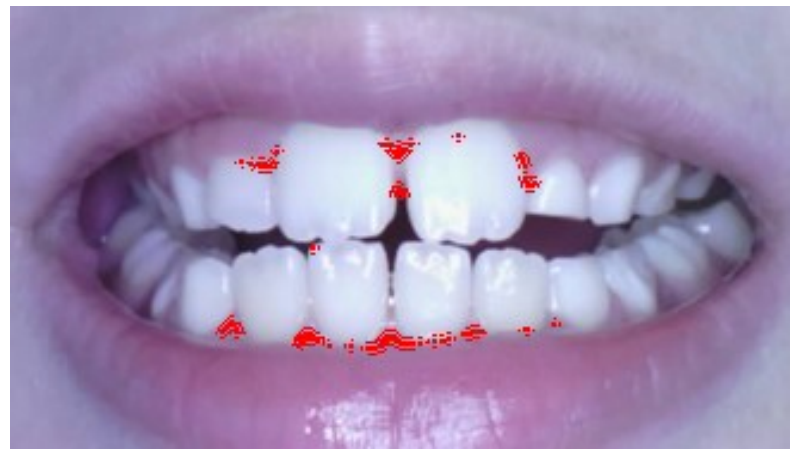	4.73	0
