# Supplementary material for: Effects of calcium-containing solutions on fluorescence-detectable plaque area and enamel mineral volume: an in vitro and exploratory clinical study
Source: BMC Oral Health. 2026 Sep 15;26:1873. doi: 10.1186/s12903-026-09789-z (PMC13628852; doi:10.1186/s12903-026-09789-z)
Supplement: Supplementary file 2 [file 12903_2026_9789_MOESM2_ESM.docx]

**Supplemental Table 1** Exploratory ANCOVA model for post-treatment QLF outcomes

| Source | Sum of Squares | df | Mean Square | F | p-value |
| --- | --- | --- | --- | --- | --- |
| Corrected Model | 37.556 | 5 | 7.511 | 7.209 | 0.006 |
| Baseline QLF | 34.894 | 1 | 34.894 | 33.49 | < 0.001 |
| Age | 6.315 | 1 | 6.315 | 6.06 | 0.036 |
| Sex | 6.567 | 1 | 6.567 | 6.302 | 0.033 |
| Treatment Group | 17.095 | 2 | 8.547 | 8.203 | 0.009 |
| Error | 9.377 | 9 | 1.042 |  |  |

Model fit: adjusted R^2^ = 0.689

For the baseline-adjusted analysis, **Post-treatment QLF** was defined as the primary dependent variable. The **Treatment Group** (Placebo, Formula 1, and Formula 2) was entered into the model as the fixed factor to assess differences in post-treatment QLF among the intervention groups. To statistically control for potential confounders and address the reviewer's concern regarding selection bias, **Baseline QLF**, **Age**, and **Sex** were incorporated as covariates. This model allowed estimation of adjusted marginal means for each group while accounting for baseline QLF, age, and sex.

**Supplemental Table 2** ANCOVA-adjusted post-treatment QLF estimated marginal means

| Treatment Group | n | Post-treatment QLF (Adjusted Mean ± SE) | 95% Confidence Interval |
| --- | --- | --- | --- |
| Group 1 (Placebo) | 5 | 4.99±0.57 | [3.70, 6.29] |
| Group 2 (Formula 1) | 5 | 2.46±0.46∗∗ | [1.43, 3.50] |
| Group 3 (Formula 2) | 5 | 1.35±0.58∗∗ | [0.03, 2.66] |

Adjusted means ± SE represent estimated marginal means from the ANCOVA model adjusted for baseline QLF, age, and sex. Estimated marginal means were evaluated at age = 6.73 years and baseline QLF = 4.73.

** *p* < 0.01 versus placebo based on post hoc pairwise comparisons of ANCOVA-adjusted estimated marginal means.

**Supplemental Table 3** Exploratory post-hoc power analysis based on change scores

| Analysis | Comparison / group | Sample size | Effect size | α | Target power | Required sample size | Actual power |
| --- | --- | --- | --- | --- | --- | --- | --- |
| Between-group change | Formula 1 vs Placebo | n = 5/group | Cohen’s d = 2.846 | 0.05 | 0.8 | n = 4/group; total n = 8 | 0.915 |
| Between-group change | Formula 2 vs Placebo | n = 5/group | Cohen’s d = 2.153 | 0.05 | 0.8 | n = 5/group; total n = 10 | 0.845 |
| One-way ANOVA change | Placebo vs Formula 1 vs Formula 2 | total n = 15; n = 5/group | Cohen’s f = 1.035 | 0.05 | 0.8 | total n = 15; 3 groups | 0.892 |
